# Banxia baizhu tianma decoction, a Chinese herbal formula, for hypertension: Integrating meta-analysis and network pharmacology

**DOI:** 10.3389/fphar.2022.1025104

**Published:** 2022-12-02

**Authors:** Jianguo Lin, Qingqing Wang, Siyu Xu, Simin Zhou, Dongsheng Zhong, Meng Tan, Xiaoxiao Zhang, Kuiwu Yao

**Affiliations:** ^1^ Guang’anmen Hospital, China Academy of Chinese Medical Sciences, Beijing, China; ^2^ Tianjin University of Traditional Chinese Medicine, Tianjin, China; ^3^ Beijing University of Chinese Medicine, Beijing, China; ^4^ Guizhou University of Traditional Chinese Medicine, Guizhou, China; ^5^ Eye Hospital China Academy of Chinese Medical Sciences, Beijing, China

**Keywords:** banxia baizhu tianma decoction, hypertension, meta-analysis, network pharmacology, blood pressure, traditional Chinese medicine

## Abstract

Hypertension is a major cardiovascular risk factor, which seriously affects the quality of life of patients. Banxia Baizhu Tianma Decoction (BXD) is a Chinese herbal formula that is widely used to treat hypertension in China. This study aimed to evaluate the efficacy and potential mechanism of BXD for hypertension by meta-analysis and network pharmacology. Meta-analysis was performed to explore the efficacy and safety of BXD combined with conventional treatment for hypertension. Network pharmacology was used to explore the molecular mechanism of BXD in antihypertension. A total of 23 studies involving 2,041 patients were included. Meta-analysis indicated that compared with conventional treatment, combined BXD treatment was beneficial to improve clinical efficacy rate, blood pressure, blood lipids, homocysteine, endothelial function, inflammation, and traditional Chinese medicine symptom score. In addition, meta-analysis indicated that BXD is safe and has no obvious adverse reactions. Network pharmacology showed that the antihypertensive targets of BXD may be AKT1, NOS3, ACE, and PPARG. The antihypertensive active ingredients of BXD may be naringenin, poricoic acid C, eburicoic acid, and licochalcone B. Due to the poor methodological quality of the Chinese studies and the small sample size of most, the analysis of this study may have been affected by bias. Therefore, the efficacy and safety of BXD for hypertension still need to be further verified by high-quality clinical studies.

**Systematic Review Registration:** https://www.crd.york.ac.uk/prospero/, identifier CRD42022353666

## 1 Introduction

Hypertension is a clinical syndrome characterized by increased systemic arterial blood pressure, which may be accompanied by functional or organic damage of the heart, brain, kidney, and other organs [Hypertension was defined as systolic blood pressure (SBP) ≥ 140 mmHg or diastolic blood pressure (DBP) ≥ 90 mmHg] (Messerli et al., 2007). Hypertension is the leading preventable risk factor for cardiovascular disease and all-cause mortality worldwide (Mills et al., 2020). The prevalence of hypertension will gradually increase with urbanization, population aging, and related lifestyle changes such as unhealthy diets and physical inactivity. In China, the prevalence of hypertension among people over 18 years old is 23.2%, which is about 240 million (Zhao et al., 2019). A 2019 study found that only 30% of people with hypertension were treated with medications, and only 10% had their blood pressure controlled below threshold levels for hypertension. High-income countries generally do better, but most have lower treatment and control rates than developed countries (Geldsetzer et al., 2019). Therefore, prevention and treatment of hypertension are urgent.

Over the past half-century, tremendous progress has been made in the pharmacological treatment of hypertension, but some shortcomings remain (adverse effects, drug resistance, long-term use, economic burden, etc.) (Bramlage and Hasford, 2009; Albasri et al., 2021). Hypertension belongs to the category of headache and vertigo in traditional Chinese medicine (TCM). TCM has a long history in the treatment of hypertension and has accumulated a lot of experience in pre-hypertension, hypertension, obese hypertension, and resistant hypertension (Xiong et al., 2013; Xiong et al., 2015; Zhang et al., 2020). Banxia Baizhu Tianma Decoction (BXD) is a TCM formula that originated from the Qing Dynasty. BXD is composed of 6 types of botanical drugs, including *Pinellia ternata* (Thunb.) Makino [Araceae] (Banxia), *Atractylodes macrocephala* Koidz. [Asteraceae] (Baizhu), *Citrus* × *aurantium* L. [Rutaceae] (Chenpi), *Glycyrrhiza uralensis* Fisch. ex DC. [Fabaceae] (Gancao), *Gastrodia elata* Blume [Orchidaceae] (Tianma), *Wolfiporia cocos* (F.A. Wolf) Ryvarden & Gilb. 1984 (Fulin). BXD has the effects of eliminating dampness and phlegm, dispelling pathogenic wind and eliminating phlegm and is widely used in hypertension and its complications (Xiong et al., 2012), however, there is a lack of high-quality, high-level evidence to further confirm its clinical efficacy. Currently, the active ingredients from BXD have been shown to have anti-inflammatory, antioxidant, vasodilator, and calcium ion regulation effects (Tan et al., 2018; Xu et al., 2022), however, the mechanism of BXD in improving hypertension has not been clarified. Network pharmacology is a new subject based on systems biology and bioinformatics, which can elucidate the mechanism of drug action at the molecular level (Xiao et al., 2021). TCM network pharmacology approach provides a new research paradigm for translating TCM from an experience-based medicine to an evidence-based medicine system, which will accelerate botanical drug discovery, and improve current drug discovery strategies (Luo et al., 2020). In this study, we aimed to validate the efficacy of BXD in hypertensive patients and to explore the underlying molecular and cellular mechanisms from network pharmacology perspective.

## 2 Materials and methods

This study was conducted and reported according to the guidelines of Preferred Reporting Items for Systematic Reviews and Meta-Analyses (PRISMA) (Liberati et al., 2009). The study protocol (CRD42022353666) was registered in the PROSPERO (https://www.crd.york.ac.uk/prospero/).

### 2.1 Literature search strategy

The databases used in this study included PubMed, Cochrane, Embase, Wanfang database, China national knowledge infrastructure (CNKI), and China Science and Technology Journal Database (VIP). The retrieval time was set as the establishment of the database until August 2022. The search terms were MeSH terms combined with the keywords: “banxia baizhu tianma” and “hypertension”. The search strategy was shown in Supplementary Table S1.

### 2.2 Inclusion and exclusion criteria

The inclusion criteria for this study were: 1) The type of study is randomized controlled trials (RCTs). 2) Patients met the diagnostic criteria for hypertension (Hypertension and League, 2019). 3) The experimental group received BXD combined with conventional treatment. The control group received conventional treatment. The treatment dose and course of treatment were unrestricted. Conventional treatment included calcium channel blockers (CCB), angiotensin-converting enzyme inhibitors (ACEI), angiotensin receptor blockers (ARBs), diuretics, and *β*-receptor blockers.

The exclusion criteria for this study were: 1) Duplicate published studies. 2) Studies with incorrect or incomplete data. 3) Unable to extract data for research. 4) Review or experiment articles.

### 2.3 Outcome measure

The primary outcome measures were: SBP, DBP, and clinical efficacy rate. The secondary outcome measures were: total cholesterol (TC), triglyceride (TG), high-density lipoprotein cholesterol (HDL-C), low-density lipoprotein cholesterol (LDL-C), homocysteine (Hcy), endothelial function, inflammatory biomarkers, and TCM symptom score.

### 2.4 Data extraction

Data were extracted independently from the included literature by SM and SY. A “basic information extraction table” was developed, and the information extracted included: the investigator, year of publication, number of cases, age, intervention, duration of intervention, and outcome measures. Any disputes were resolved through discussion with the third author (QW). When necessary, study details were requested from the corresponding authors *via* email.

### 2.5 Risk of bias and quality assessment

The quality of the included studies was evaluated with the risk bias assessment by Cochrane collaboration’s tool (Higgins et al., 2011), including random sequence generation, allocation hiding, blinding of practitioners and subjects, blinding of outcome evaluators, the integrity of outcome data, selective reporting of results, and other sources of bias. Three evaluation results, namely low risk, high risk, and unclear risk, were made one by one.

### 2.6 Statistical analysis

The Stata 17.0 software (Stata Corp., College Station, TX, United States) was applied to statistical analysis. Standardized mean difference (SMD) was utilized for continuous outcomes. Risk ratio (RR) was utilized for dichotomous outcomes. All of them were expressed with a 95% confidence interval (CI). Heterogeneity was tested using the Q test, and if *I* ^2^ ≤ 50%, a fixed-effects model was used, and if *I* ^2^ > 50%, indicating greater statistical heterogeneity, a random-effects model was used. Both results were expressed using a forest plot. The publication bias was estimated by Egger’s test and funnel plot. It was regarded as a significant difference when *p* < 0.05.

### 2.7 Identify BXD and hypertension targets

With the Traditional Chinese Medicine Systems Pharmacology Database and Analysis Platform (TCMSP, https://tcmspw.com) (Ru et al., 2014), the active ingredients of BXD were obtained. The TCMSP parameter was set as bioavailability (OB) ≥ 30% and drug-like properties (DL) ≥ 0.18 (Guo et al., 2019). The targets corresponding to the active ingredients were obtained by using the Swiss Target Prediction database (http://swisstargetprediction.ch/). With “hypertension” as the keyword, the targets of hypertension were obtained through four databases. The four different databases and search criteria are as follows: GeneCards (https://www.genecards.org/) (Stelzer et al., 2016), and the screening criterion is relevance score ≥ 4; Comparative Toxicogenomics Database (CTD) (https://ctdbase.org/) (Davis et al., 2019), and the screening criterion is direct evidence or inference score ≥ 100; DisGeNET (https://www.disgenet.org) (Piñero et al., 2020), and the screening standard is gene-disease association score ≥ 0.2. INPUT 2.0 (http://cbcb.cdutcm.edu.cn/INPUT/) (Li Q et al., 2022), and the screening criterion set the default parameters. Subsequently, the intersection of these targets was taken to obtain the crossover targets.

### 2.8 Protein-protein interaction and gene enrichment analysis

PPI analysis of overlapping targets was conducted through STRING platform (https://string-db.org/) (Szklarczyk et al., 2019), and the calculated results were imported into Cytoscape 3.9.1 software (Shannon et al., 2003) for network topology analysis. CytoHubba plug-in was used to screen key targets. Gene enrichment of overlapping targets was performed through DAVID database (https://david.ncifcrf.gov/) (Huang et al., 2009). It mainly includes molecular function (MF), cellular components (CC), biological process (BP), and kyoto encyclopedia of genes and genomes (KEGG). Hiplot (https://hiplot-academic.com/) was used to visualize the results.

### 2.9 Molecular docking

Selected key targets and key ingredients for molecular docking. The key target structures were obtained through PDB database (https://www.rcsb.org/). The structure of the key ingredients was obtained through the PubChem database (https://pubchem.ncbi.nlm.nih.gov/). Then used Pymol, Autodock Vina (Trott and Olson, 2010), and PLIP (https://plip-tool.biotec.tu-dresden.de/) (Adasme et al., 2021) for molecular docking.

## 3 Results

### 3.1 Eligible studies

**FIGURE 1:**
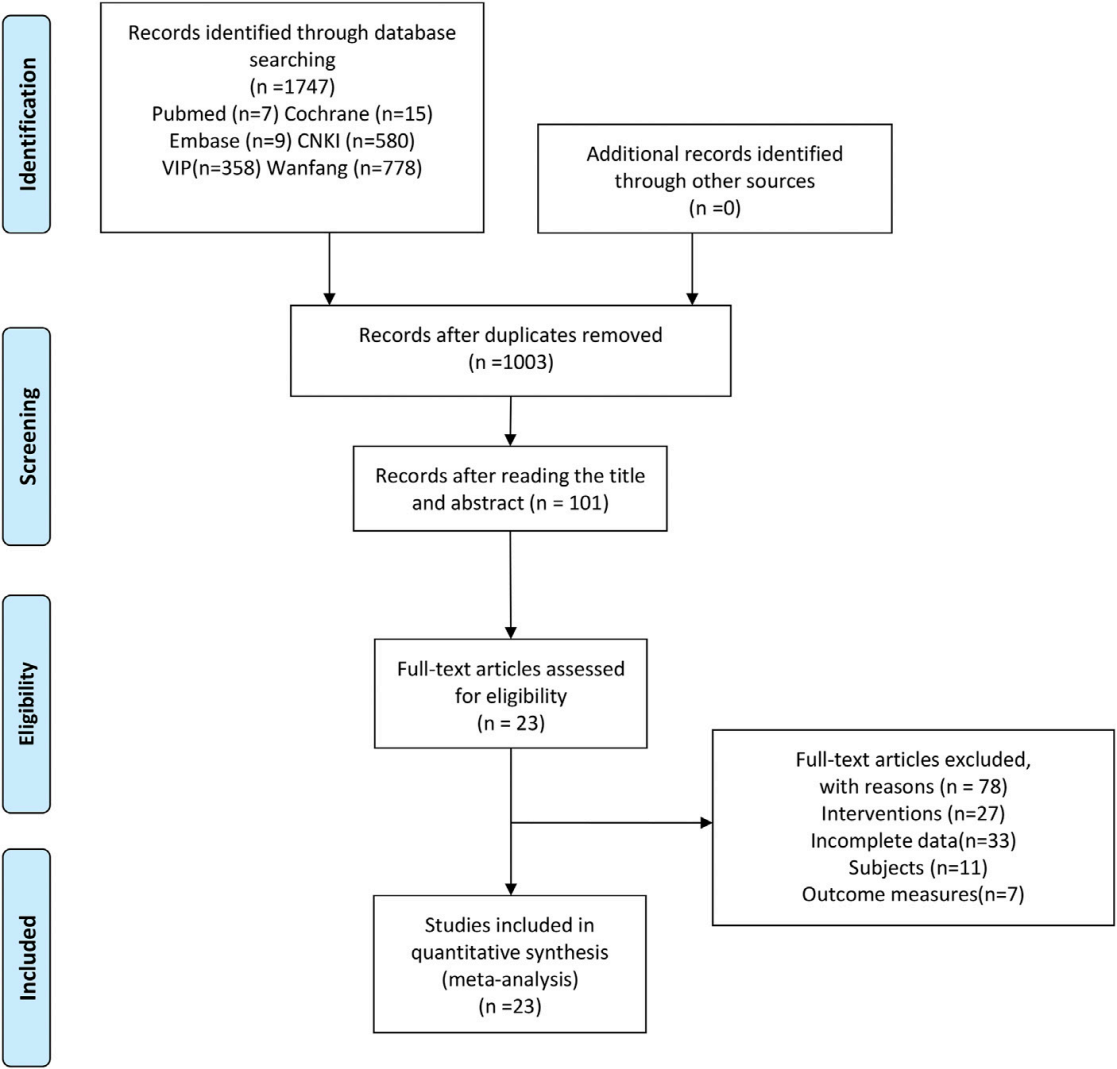
Flow diagram of the study selection process.

A total of 1,747 related studies were retrieved. 1,003 studies remain after the elimination of duplicates by NoteExpress software and manual assistance. After reading the abstract and title, 101 studies remain. After reading the full text, 78 studies were excluded. Finally, a total of 23 studies were included (Wu et al., 2007; Xiong, 2010; Pang, 2013; Huang and Li, 2014; Shen and Jin, 2015; Guan and Chen, 2016; Liu, 2016; Wu and Zhou, 2016; Zhao et al., 2016; Miao et al., 2017; Song, 2018; Ma et al., 2019; Shi, 2019; Wu, 2019; Liu et al., 2020; Mu, 2020; Tang et al., 2020; Zhang, 2021; Zhang et al., 2021; Zheng, 2021; Dai et al., 2022; Zhang et al., 2022; Zhao, 2022). The flow diagram of screening was shown in Figure 1.

### 3.2 Characteristics of studies

A total of 23 RCTs involving 2,041 patients (treatment group: 1,025, control group: 1,016) were included. The publication period was from 2007 to 2022. The shortest treatment time was 4 weeks, and the longest treatment time was 12 weeks. Types of hypertension include H-type hypertension (Pang, 2013; Wu and Zhou, 2016; Liu et al., 2020; Zhao, 2022), senile hypertension (Liu et al., 2020; Mu, 2020; Zhang, 2021), hypertensive crisis (Zhang et al., 2021), obesity type hypertension (Huang and Li, 2014; Zhao et al., 2016; Shi, 2019), phlegm-dampness type hypertension (Wu et al., 2007; Xiong, 2010; Huang and Li, 2014; Guan and Chen, 2016; Zhao et al., 2016; Miao et al., 2017; Shi, 2019; Liu et al., 2020; Mu, 2020; Tang et al., 2020; Zhang et al., 2021; Dai et al., 2022; Zhang et al., 2022). In all the studies, BXD was the basic prescription, and botanical drugs (Such as *Alisma plantago-aquatica* L. [Alismataceae] (Zexie), *Neolitsea cassia* (L.) Kosterm. [Lauraceae] (Guizhi), *Arisaema heterophyllum* Blume [Araceae] (Dannanxing), *Conioselinum anthriscoides* ‘*Chuanxiong*’ [Apiaceae] (Chuanxiong), *Chrysanthemum* × *morifolium* (Ramat.) Hemsl. [Asteraceae] (Juhua), *Vitex trifolia* L. [Lamiaceae] (Manjingzi), *Acorus gramineus* Aiton [Acoraceae] (Shichangpu)) were added or subtracted according to syndrome differentiation. The characteristics of the included studies were shown in Table 1. The composition of the prescriptions was shown in Supplementary Table S2.

**TABLE 1:** Characteristics of the included studies.

Study	Sample size (T/C)	Mean age (years) (T/C)	Intervention (T/C)	Duration	Outcome measures
Dai et al. (2022)	45/45	51.39 ± 6.38	BXD + CT/CT	8 weeks	①②⑤
		53.78 ± 7.61			
Zhang et al. (2022)	54/54	55.19 ± 16.73	BXD + CT/CT	12 weeks	①⑤⑦
		56.51 ± 15.41			
Zhao, (2022)	45/45	65.77 ± 8.23	BXD + CT/CT	8 weeks	②④
		66.12 ± 8.40			
Zhang, (2021)	36/36	72.2 ± 2.1	BXD + CT/CT	6 weeks	①②
		72.8 ± 2.3			
Zheng, (2021)	54/54	55.12 ± 5.87	BXD + CT/CT	4 weeks	②
		55.14 ± 3.27			
Zhang et al. (2021)	40/40	52.74 ± 7.71	BXD + CT/CT	4 weeks	①②⑦
		53.11 ± 8.99			
Liu et al. (2020)	49/48	NA	BXD + CT/CT	8 weeks	②④
Tang et al. (2020)	43/43	56.84 ± 7.69	BXD + CT/CT	4 weeks	①②
		56.84 ± 7.69			
Mu, (2020)	44/44	77.20 ± 4.18	BXD + CT/CT	4 weeks	①②⑤
		76.48 ± 3.62			
Ma et al. (2019)	100/100	54.24 ± 12.6	BXD + CT/CT	4 weeks	①②③⑥
		54.14 ± 12.57			
Wu, (2019)	30/30	52.68 ± 5.25	BXD + CT/CT	4 weeks	②④⑦
		51.45 ± 4.99			
Shi, (2019)	62/61	53.86 ± 8.37	BXD + CT/CT	8 weeks	②③
		52.71 ± 8.12			
Song, (2018)	32/32	57.46 ± 11.29	BXD + CT/CT	8 weeks	①②⑥
		56.85 ± 11.08			
Miao et al. (2017)	44/44	56.52 ± 6.35	BXD + CT/CT	4 weeks	②
Zhao et al. (2016)	40/40	62.34 ± 9.32	BXD + CT/CT	12 weeks	①②③
		64.18 ± 8.67			
Guan and Chen, (2016)	30/30	63.5 ± 6.8	BXD + CT/CT	4 weeks	①②③④
		62.9 ± 6.5			
Liu, (2016)	43/43	48.97 ± 6.24	BXD + CT/CT	4 weeks	②④
		51.08 ± 7.05			
Wu and Zhou, (2016)	50/50	62.8 ± 6.1	BXD + CT/CT	8 weeks	②④⑥
		63 ± 5.8			
Shen and Jin, (2015)	30/30	55.13 ± 12.05	BXD + CT/CT	8 weeks	①②⑥
		54.27 ± 11.37			
Huang and Li, (2014)	50/48	58.7 ± 1.8	BXD + CT/CT	4 weeks	①②
		59.4 ± 1.1			
Pang, (2013)	30/26	63.4 ± 6.6	BXD + CT/CT	4 weeks	①②④
		62.8 ± 6.5			
Xiong, (2010)	30/30	53.87 ± 5.92	BXD + CT/CT	4 weeks	①②
		52.87 ± 5.4			
Wu et al. (2007)	44/43	53.6 ± 8	BXD + CT/CT	8 weeks	②③
		52.8 ± 7.3			

Note: T: treatment group; C: control group; BXD: banxia baizhu tianma decoction; CT: Conventional treatment (including CCB, ACEI, ARB, diuretics, and *β*-receptor blocker); ①Clinical efficacy rate; ②Blood pressure; ③Blood lipids; ④Hcy; ⑤Endothelial function; ⑥Inflammatory biomarkers; ⑦TCM, symptom score.

### 3.3 Risk of bias and quality assessment of studies

**FIGURE 2:**
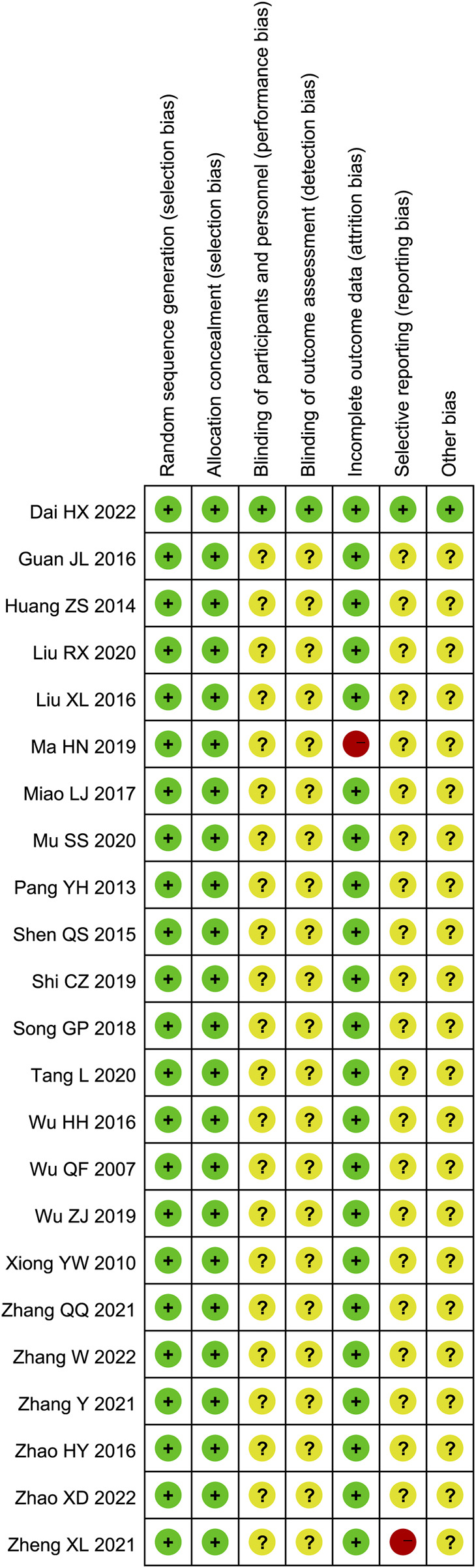
Risk of bias assessments of included studies.

All studies were randomized, and the majority of studies described specific randomization methods. One study (Dai et al., 2022) described the implementation of blinding. One study (Liu et al., 2020) did not describe specific baseline characteristics. Overall, the quality of the studies was not high. The risk of bias in the included studies were presented in Figure 2.

### 3.4 Meta-analysis results

The calculated results of all meta-analyses were shown in Table 2.

**TABLE 2:** Calculation results of the meta-analysis.

Outcome measures	Trials	Sample size	SMD/RR	95% CI	*Z*	*P*	*I* ^2^ (%)	*P* For heterogeneity
Clinical efficacy rate	14	1,202	1.25	(1.19, 1.32)	8.72	0.00	0.00	0.78
SBP	22	1933	−1.21	(−1.56, −0.86)	−6.78	0.00	91.81	0.00
DBP	22	1933	−1.01	(−1.33, −0.69)	−6.22	0.00	90.76	0.00
TC	5	550	−0.70	(−1.11, −0.28)	−3.30	0.00	81.07	0.00
TG	5	550	−0.80	(−1.46, −0.14)	−2.37	0.02	92.50	0.00
LDL-C	5	550	−0.58	(−0.75, −0.41)	−6.62	0.00	0.00	0.49
HDL-C	5	550	0.51	(0.34, 0.68)	5.84	0.00	47.43	0.11
Hcy	7	549	−2.26	(−3.43, −1.09)	−3.79	0.00	96.56	0.00
NO	3	286	0.88	(0.63, 1.12)	7.09	0.00	0.00	0.99
ET-1	3	286	−1.09	(−1.33, −0.84)	−8.57	0.00	0.00	0.79
CRP	4	284	−2.19	(−3.30, −1.09)	−3.88	0.00	92.66	0.00
IL-6	3	184	−2.65	(−4.67, −0.63)	−2.57	0.01	95.87	0.00
Vertigo	3	124	−2.57	(−4.09, −1.04)	−3.29	0.00	94.96	0.00
Anorexia	3	124	−2.95	(−4.63, −1.27)	−3.44	0.00	95.21	0.00
Chest tightness and fatigue	3	124	−2.77	(−4.91, −0.62)	−2.53	0.01	97.31	0.00
Adverse reactions	13	1,273	0.52	(0.35, 0.78)	−3.20	0.00	0.00	0.91

#### 3.4.1 Clinical efficacy rate

**FIGURE 3:**
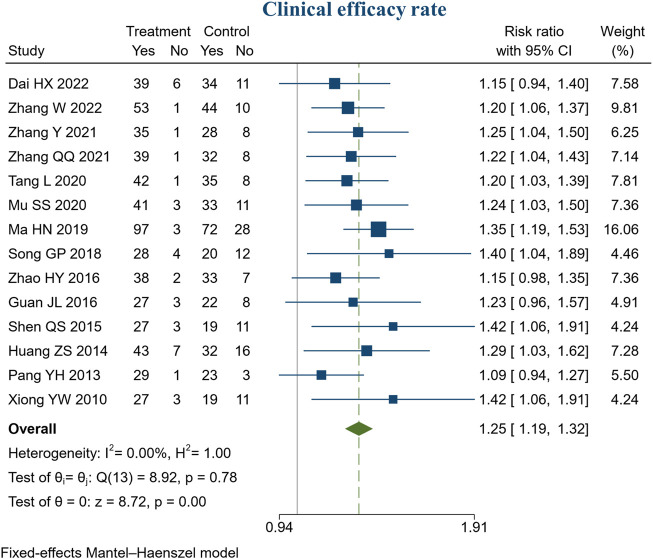
Forest plot of clinical efficacy rate.

14 studies reported clinical efficacy rate. There were 604 patients in the treatment group and 598 in the control group. Meta-analysis indicated that BXD combined with conventional treatment had a higher clinical efficacy rate than conventional treatment (RR = 1.25, 95% CI [1.19, 1.32], *I* ^2^ = 0%, *p* < 0.05, Figure 3).

#### 3.4.2 Blood pressure

**FIGURE 4:**
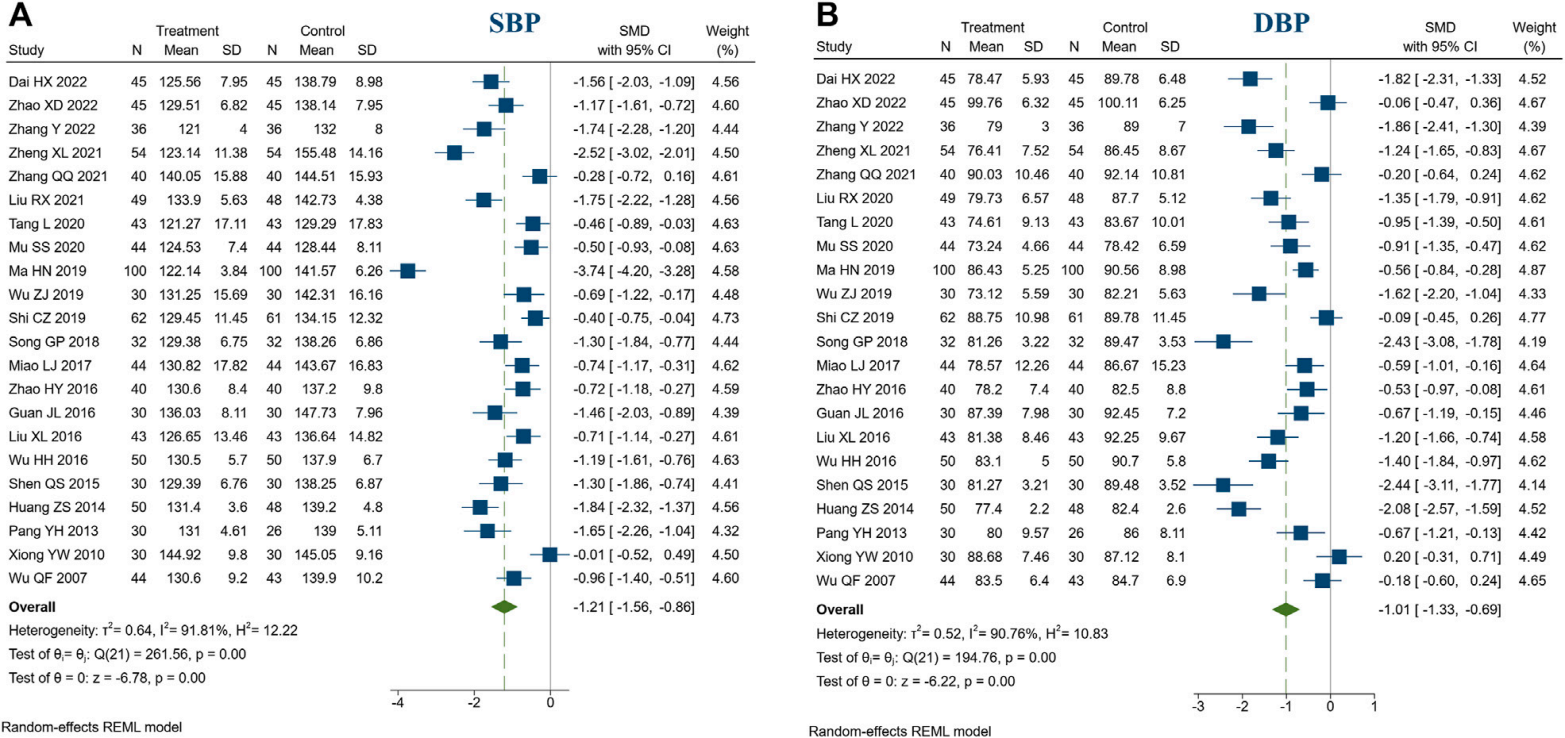
Forest plot of blood pressure. **(A)** SBP; **(B)** DBP.

22 studies reported blood pressure. There were 971 patients in the treatment group and 962 in the control group. Meta-analysis indicated that BXD combined with conventional treatment had a better ability to lower SBP than conventional treatment (SMD = −1.21, 95% CI [−1.56, −0.86], *I* ^2^ = 91.81%, *p* < 0.05, Figure 4A). Sensitivity analysis indicated that the heterogeneity might be caused by 3 studies (Xiong, 2010; Ma et al., 2019; Zheng, 2021), and heterogeneity was reduced by excluding these studies (*I* ^2^ = 78.27%, Supplementary Figure S1). In addition, meta-analysis indicated that BXD combined with conventional treatment had a better ability to lower DBP than conventional treatment (SMD = −1.01, 95% CI [−1.33, −0.69], *I* ^2^ = 90.76%, *p* < 0.05, Figure 4B). We speculate that heterogeneity resulted from the use of the post-intervention mean (Supplementary Figure S2).

#### 3.4.3 Blood lipids

**FIGURE 5:**
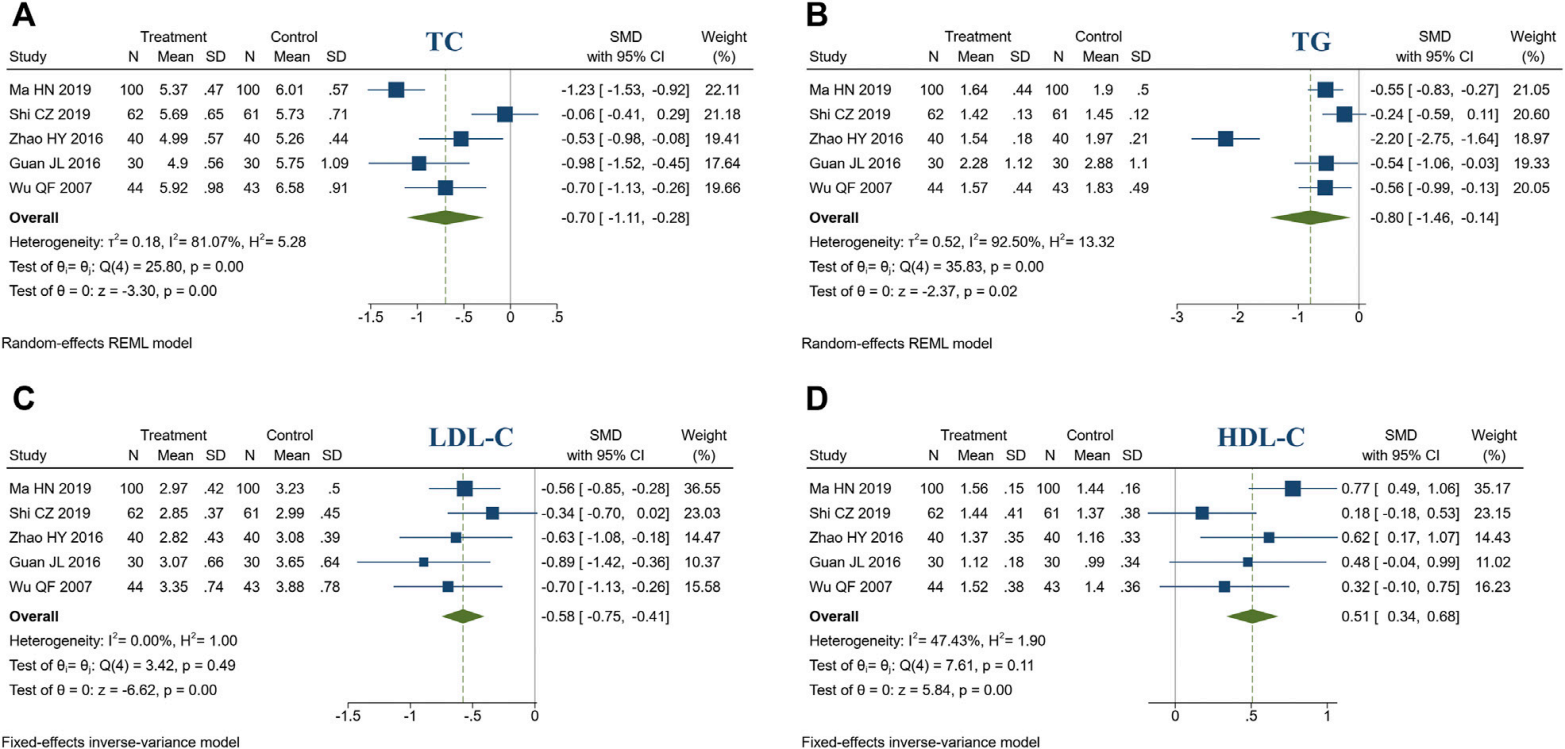
Forest plot of blood lipids. **(A)** TC; **(B)** TG; **(C)** LDL-C; **(D)** HDL-C.

5 studies reported blood lipids. There were 276 patients in the treatment group and 274 in the control group. Meta-analysis indicated that compared to the control group, treatment group had a better ability to improve TC(SMD = −0.70, 95% CI [−1.11, −0.28], *I* ^2^ = 81.07%, *p* < 0.05, Figure 5A), TG (SMD = -0.80, 95% CI [-1.46, -0.14], *I* ^2^ = 92.50%, *p* < 0.05, Figure 5B), LDL-C (SMD = −0.58, 95% CI [−0.75, -0.41], *I* ^2^ = 0%, *p* < 0.05, Figure 5C), HDL-C (SMD = 0.51, 95% CI [0.34, 0.68], *I* ^2^ = 47.43%, *p* < 0.05, Figure 5D). Sensitivity analysis indicated that the heterogeneity of TC might be caused by 2 studies (Ma et al., 2019; Shi, 2019), and heterogeneity was reduced by excluding these studies (*I* ^2^ = 0%, Supplementary Figure S3). Sensitivity analysis indicated that the heterogeneity of TG might be caused by 1 study (Zhao et al., 2016), and heterogeneity was reduced by excluding the studies (*I* ^2^ = 0%, Supplementary Figure S4). In addition, we speculated that the heterogeneity was due to different ways of measuring blood lipids.

#### 3.4.4 Homocysteine

**FIGURE 6:**
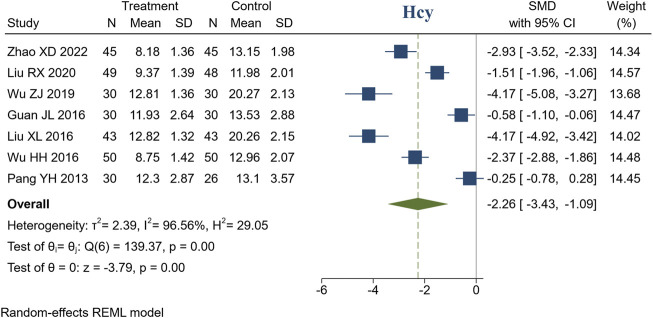
Forest plot of Hcy.

7 studies reported Hcy. There were 277 patients in the treatment group and 272 in the control group. Meta-analysis indicated that compared to the control group, the treatment group had a better ability to decrease Hcy (SMD = −2.26, 95% CI [−3.43, −1.09], *I* ^2^ = 96.56%, *p* < 0.05, Figure 6). Sensitivity analysis indicated that the heterogeneity may be caused by different measurement methods of Hcy (Supplementary Figure S5).

#### 3.4.5 Endothelial function

**FIGURE 7:**
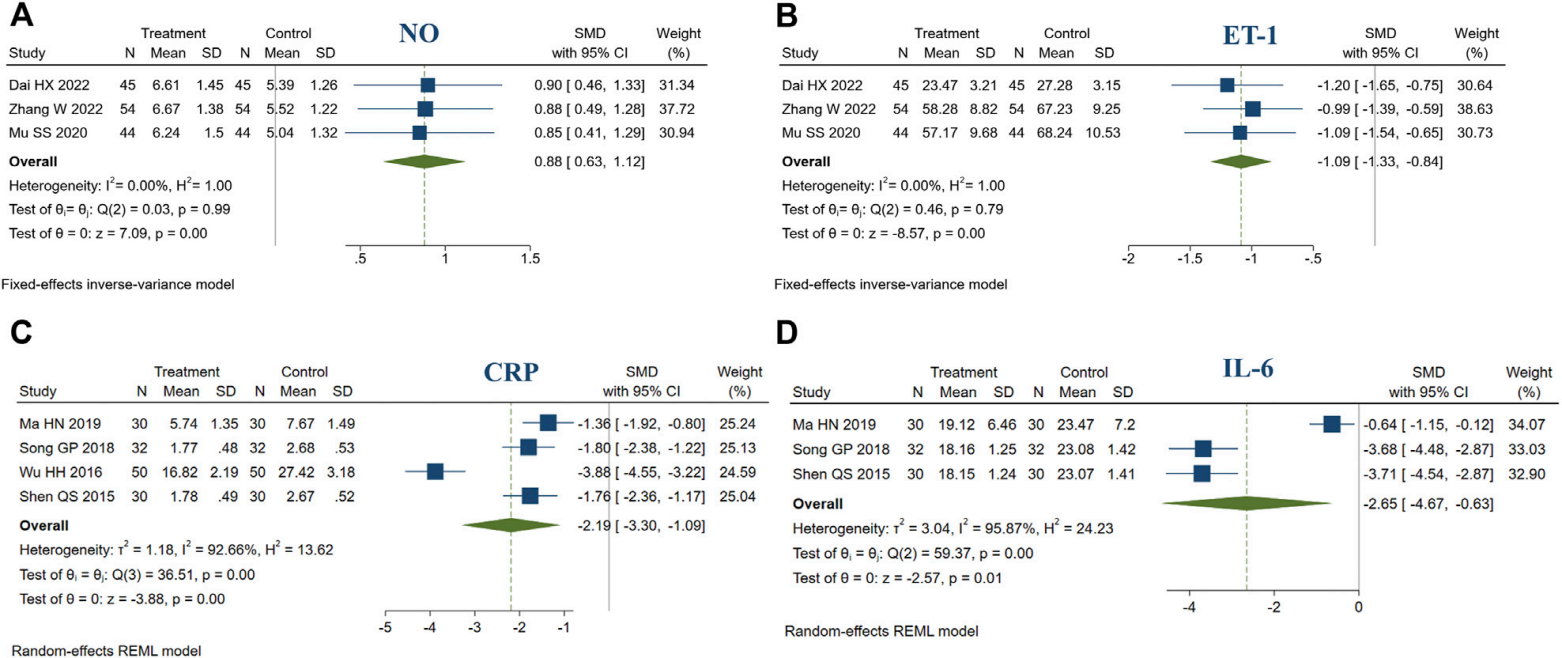
Forest plot of endothelial function and inflammatory biomarkers. **(A)** NO; **(B)** ET-1; **(C)** CRP; **(D)** IL-6.

3 studies reported endothelial function (NO, ET-1). There were 143 patients in the treatment group and 143 in the control group. Meta-analysis indicated that compared to the control group, the treatment group had a better ability to increase NO (SMD = 0.88, 95% CI [0.63, 1.12], *I* ^2^ = 0%, *p* < 0.05, Figure 7A). Similarly, compared to the control group, treatment group had a better ability to decrease ET-1 (SMD = −1.09, 95% CI [−1.33, −0.84], *I* ^2^ = 0%, *p* < 0.05, Figure 7B).

#### 3.4.6 Inflammatory biomarkers

4 studies reported CRP. There were 142 patients in the treatment group and 142 in the control group. Meta-analysis indicated that compared to the control group, the treatment group had a better ability to decrease CRP (SMD = −2.19, 95% CI [−3.30, −1.09], *I* ^2^ = 92.66%, *p* < 0.05, Figure 7C). In addition, 3 studies reported IL-6. There were 92 patients in the treatment group and 92 in the control group. Meta-analysis indicated that compared to the control group, the treatment group had a better ability to decrease IL-6 (SMD = −2.65, 95% CI [−4.67, −0.63], *I* ^2^ = 95.87%, *p* < 0.05, Figure 7D). Sensitivity analysis suggested that the heterogeneity was caused by the different measurement methods and units of IL-6 and CRP (Supplementary Figures S6, S7).

#### 3.4.7 TCM symptom scores

**FIGURE 8:**
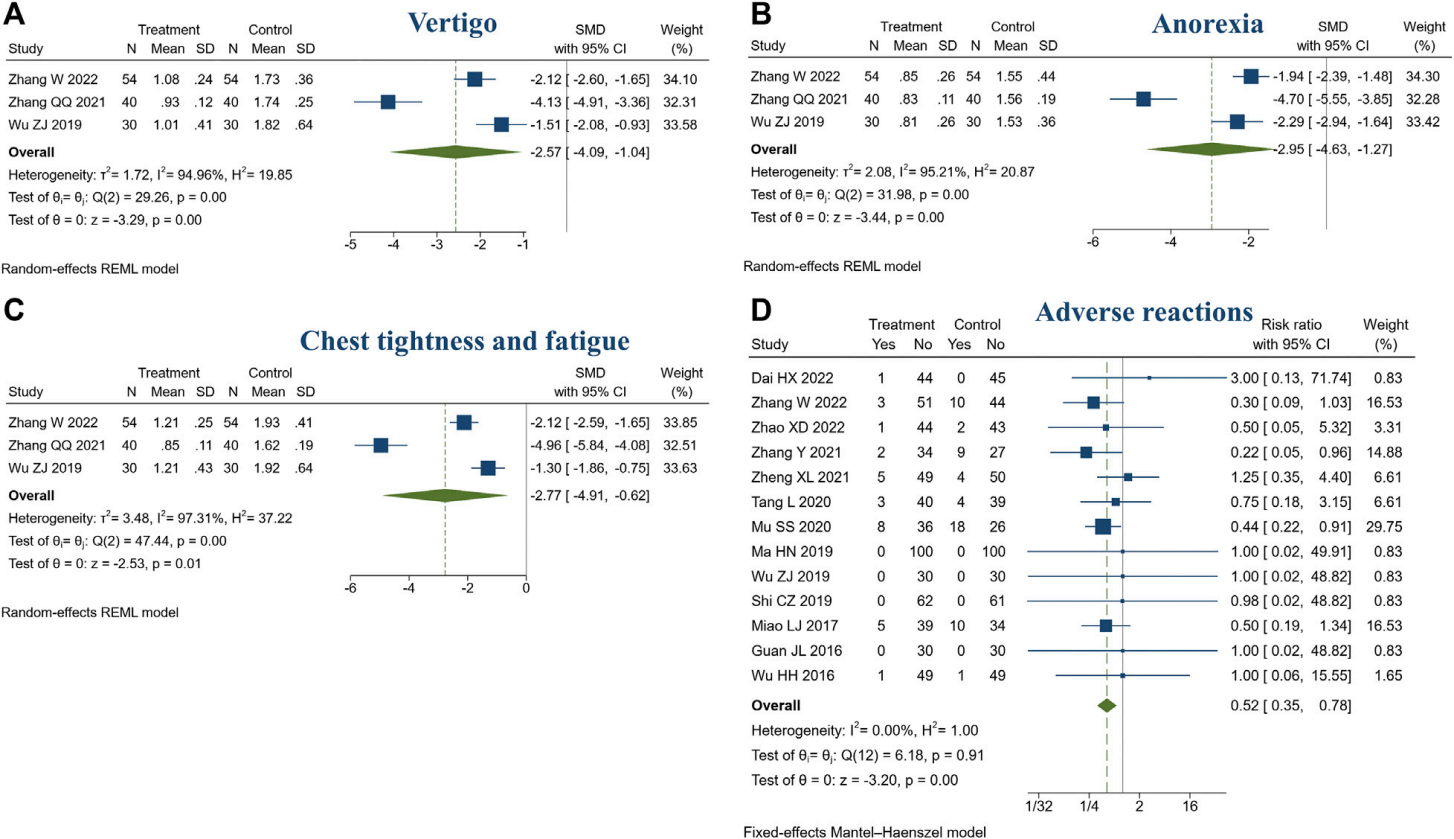
Forest plot of TCM symptom score and adverse reactions. **(A)** Vertigo; **(B)** Anorexia; **(C)** Chest tightness and fatigue; **(D)** Adverse reactions.

3 studies reported TCM symptom scores. There were 124 patients in the treatment group and 124 in the control group. Meta-analysis showed that compared with the control group, the treatment group had a stronger ability to improve the TCM symptoms, such as vertigo (SMD = −2.57, 95% CI [−4.09, −1.04], *I* ^2^ = 94.96%, *p* < 0.05, Figure 8A), anorexia (SMD = −2.95, 95% CI [−4.63, −1.27], *I* ^2^ = 95.21%, *p* < 0.05, Figure 8B), chest tightness and fatigue (SMD = −2.77, 95% CI [−4.91, −0.62], *I* ^2^ = 97.31%, *p* < 0.05, Figure 8C). Sensitivity analysis suggested that different TCM syndrome score evaluation criteria may be the cause of heterogeneity (Supplementary Figures S8, S9, S10).

#### 3.4.8 Adverse reactions

In this review, 14 studies reported adverse reactions (14/23, 60.87%) (Huang and Li, 2014; Guan and Chen, 2016; Wu and Zhou, 2016; Miao et al., 2017; Ma et al., 2019; Shi, 2019; Wu, 2019; Mu, 2020; Tang et al., 2020; Zhang, 2021; Zheng, 2021; Dai et al., 2022; Zhang et al., 2022; Zhao, 2022). Among them, no adverse reactions were identified in both treatment or control groups (Guan and Chen, 2016; Ma et al., 2019; Shi, 2019; Wu, 2019). One study did not specifically describe it (Huang and Li, 2014). The adverse reactions included nausea, vomiting, headache, palpitation, loss of appetite, diarrhea, sweating, etc. The symptoms were mild, tolerable, and could be relieved automatically. Meta-analysis showed the treatment group was safer than the control group (RR = 0.52, 95% CI [0.35, 0.78], *I* ^2^ = 0%, *p* < 0.05, Figure 8D).

#### 3.4.9 Publication bias

**FIGURE 9:**
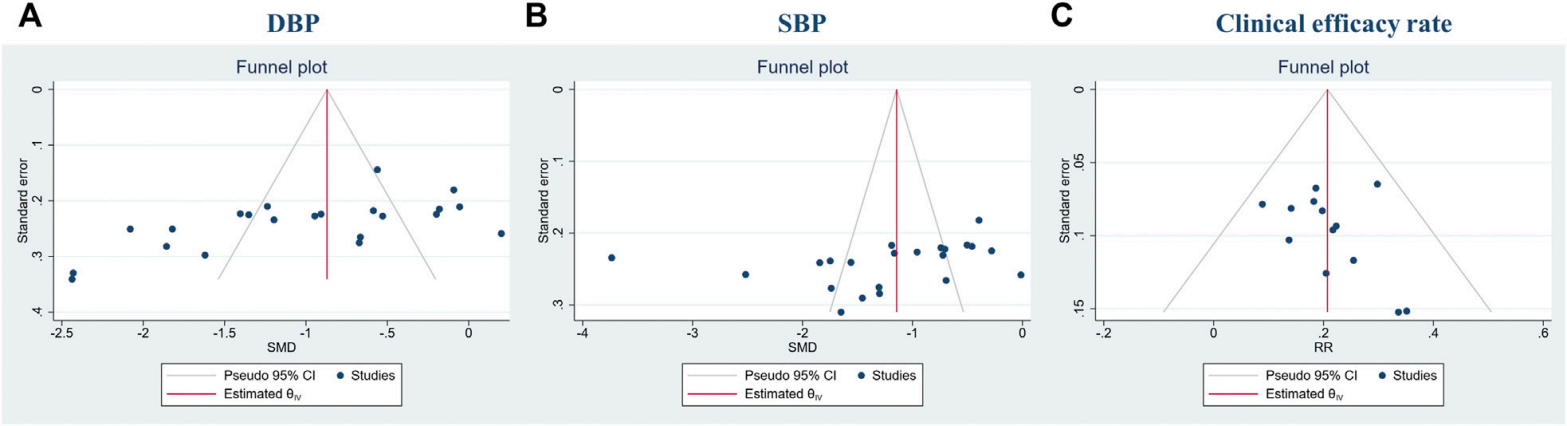
Funnel plot. **(A)** DBP; **(B)** SBP; **(C)** Clinical efficacy rate.

SBP, DBP, and clinical efficacy rate were evaluated by publication bias. The funnel plot and Egger’s test suggested the possibility of publication bias in DBP (Egger’s test: *p* < 0.05, Figure 9A). SBP and clinical efficacy rate did not show publication bias (Egger’s test: *p* = 0.1383, *p* = 0.2814, Figures 9B,C).

### 3.5 Analysis of BXD and hypertension targets

**FIGURE 10:**
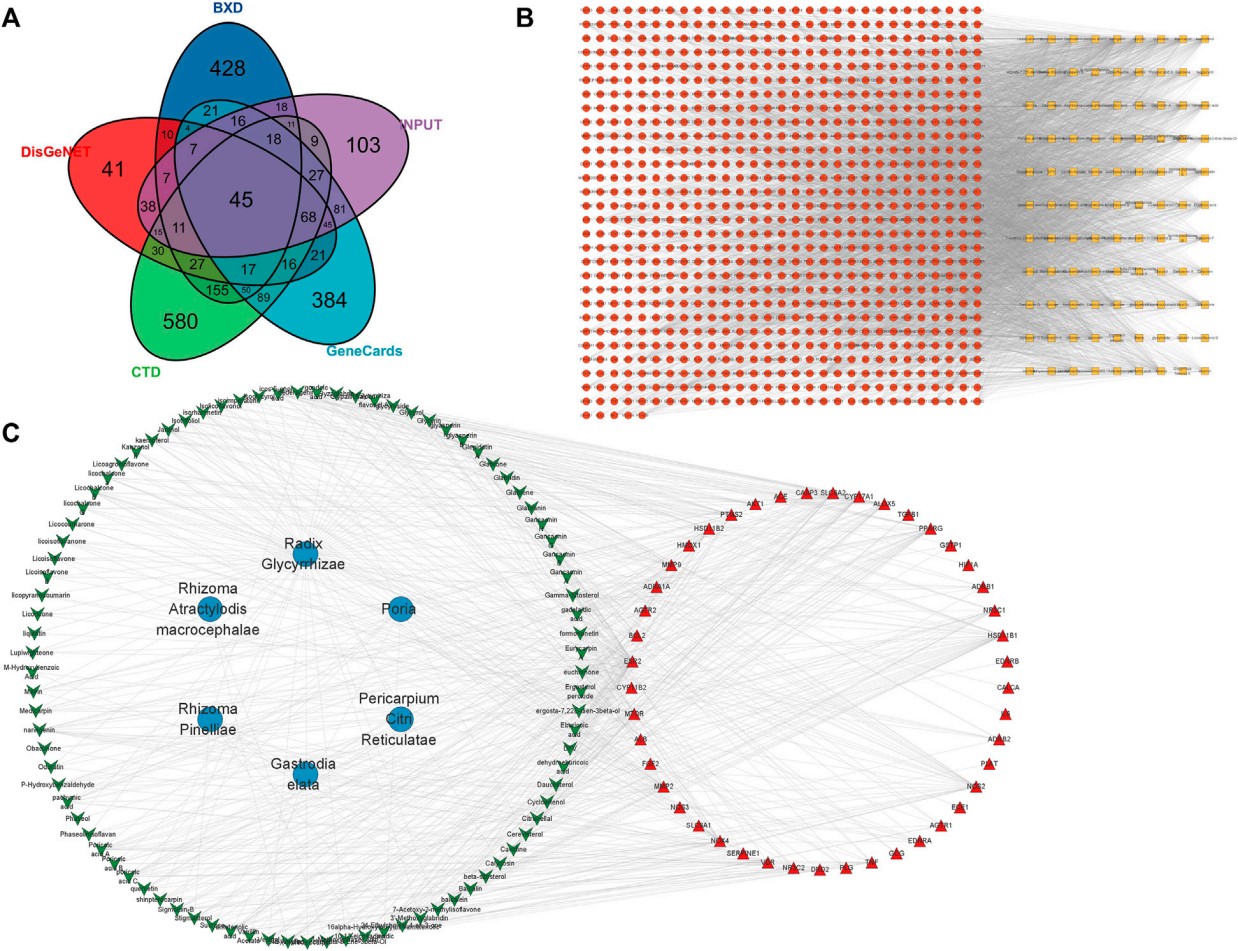
Network pharmacology of BXD and hypertension. **(A)** VENN of hypertension and BXD; **(B)** BXD-target network **(C)** BXD and hypertension network.

A total of 99 active ingredients and 845 targets corresponding to the active ingredients were collected. A total of 45 intersection targets of hypertension and BXD were obtained (Figure 10A). After that, the BXD-target network was constructed by Cytoscape (Figure 10B). Finally, the BXD and hypertension network was constructed (Figure 10C) and the key active ingredients were screened by analyzing network function. The list of top 20 active ingredients was listed according to the degree (Table 3).

**TABLE 3:** Top 20 active ingredients.

Degree	Name
16	16alpha-Hydroxydehydrotrametenolic acid
15	Naringenin
14	Eburicoic acid
14	Poricoic acid C
13	Gondoic acid
13	Trametenolic acid
13	Poricoic acid A
12	Hederagenin
12	Icos-5-enoic acid
12	Gadelaidic acid
11	Dehydroeburicoic acid
10	Cerevisterol
10	Pachymic acid
10	Sigmoidin-B
10	Jaranol
10	Licochalcone B
9	Glypallichalcone
9	Cavidine
9	24-Ethylcholest-4-en-3-one
9	Glepidotin A

### 3.6 PPI and gene enrichment analysis

**FIGURE 11:**
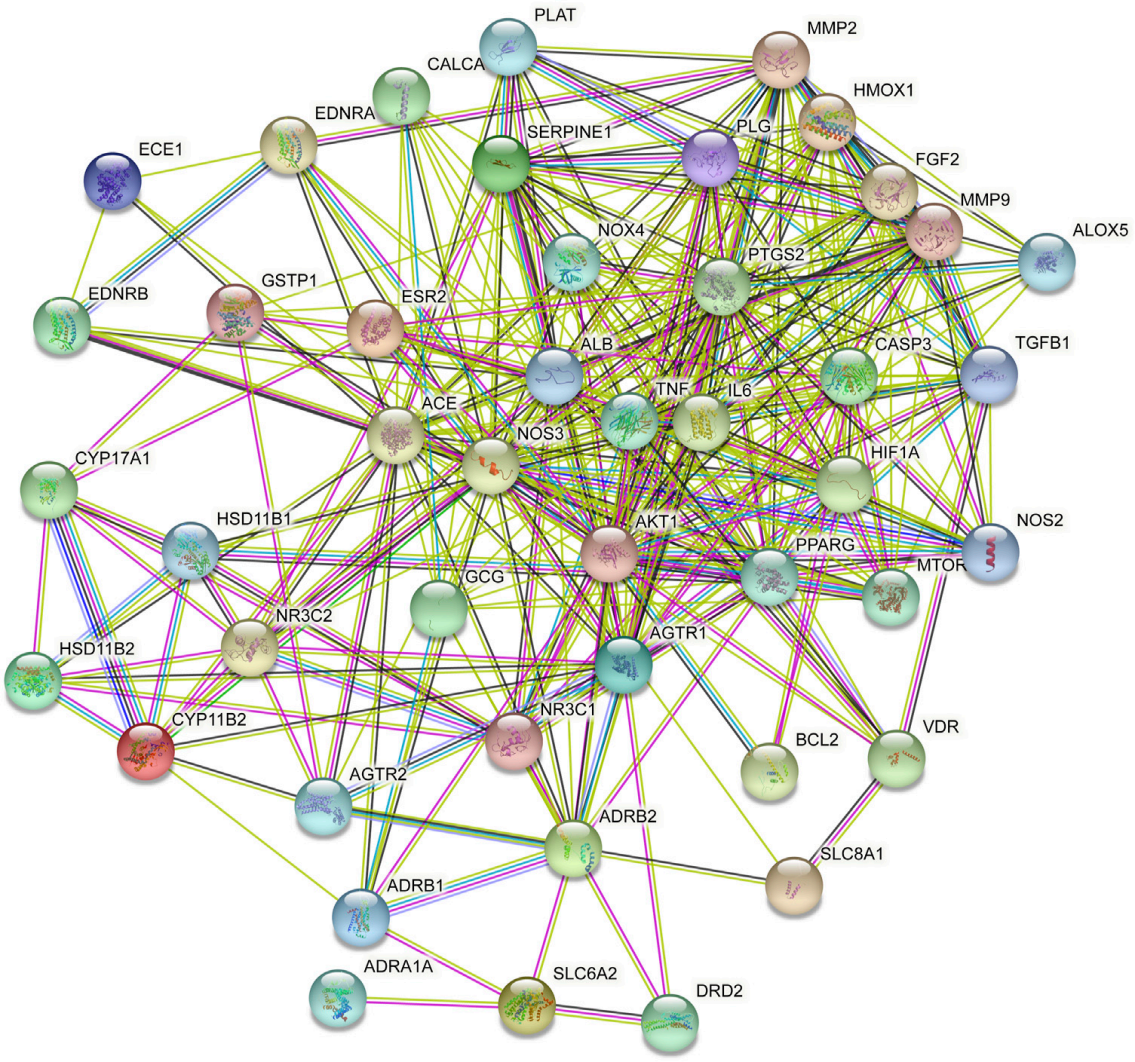
PPI of the key target.

**FIGURE 12:**
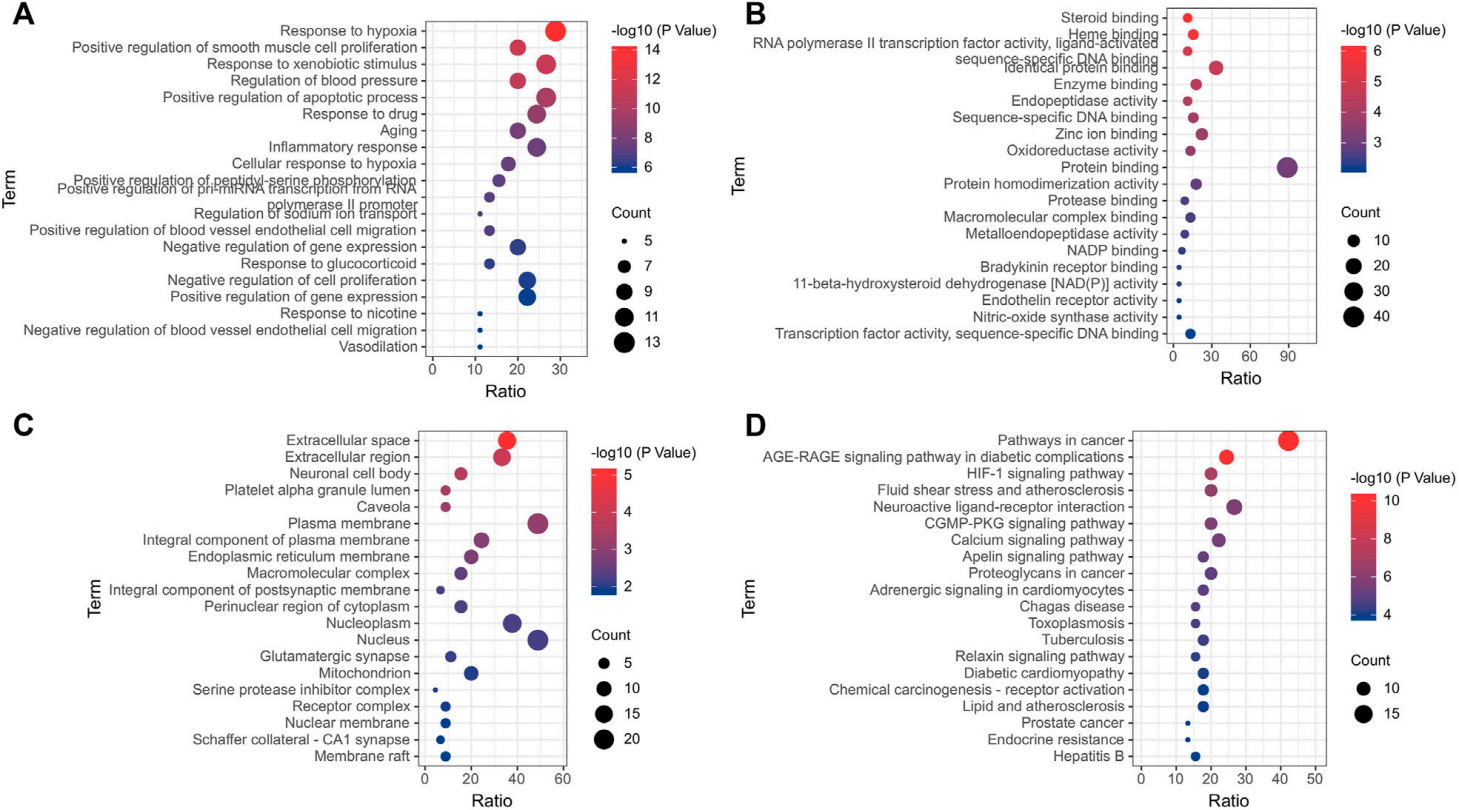
Enrichment analysis of gene. **(A)** Biological process; **(B)** Molecular function; **(C)** Cellular components; **(D)** KEGG.

PPI processing was conducted on the overlapping targets through STRING platform (Figure 11) and using Cytoscape to screen key targets. The key targets were NOS3, ACE, AKT1, TNF, ALB, PPARG, PTGS2, and CASP3 (Table 4). Gene enrichment analysis was conducted on the overlapping targets through DAVID. The results showed that the main BP items were response to hypoxia, positive regulation of smooth muscle cell proliferation, response to xenobiotic stimulus, regulation of blood pressure, inflammatory response, positive regulation of apoptotic process, positive regulation of blood vessel endothelial cell migration (Figure 12A). The main MF items were steroid binding, heme binding, identical protein binding, endopeptidase activity, and oxidoreductase activity (Figure 12B). The main CC items were extracellular space, extracellular region, neuronal cell body, platelet alpha granule lumen, and caveola. (Figure 12C). Visualization of the top 20 KEGG pathways (Figure 12D), involving AGE-RAGE signaling pathway in diabetic complications, HIF-1 signaling pathway fluid shear stress and atherosclerosis, cGMP-PKG signaling pathway, calcium signaling pathway, adrenergic signaling in cardiomyocytes.

**TABLE 4:** Top 20 key targets.

Degree	Name
33	NOS3
32	ACE
32	AKT1
31	TNF
31	ALB
30	IL6
26	PPARG
25	PTGS2
24	CASP3
23	MMP9
22	HIF1A
21	MMP2
21	TGFB1
21	SERPINE1
20	HMOX1
20	AGTR1
18	FGF2
17	PLG
16	MTOR
15	NOX4

### 3.7 Analysis of molecular docking

**FIGURE 13:**
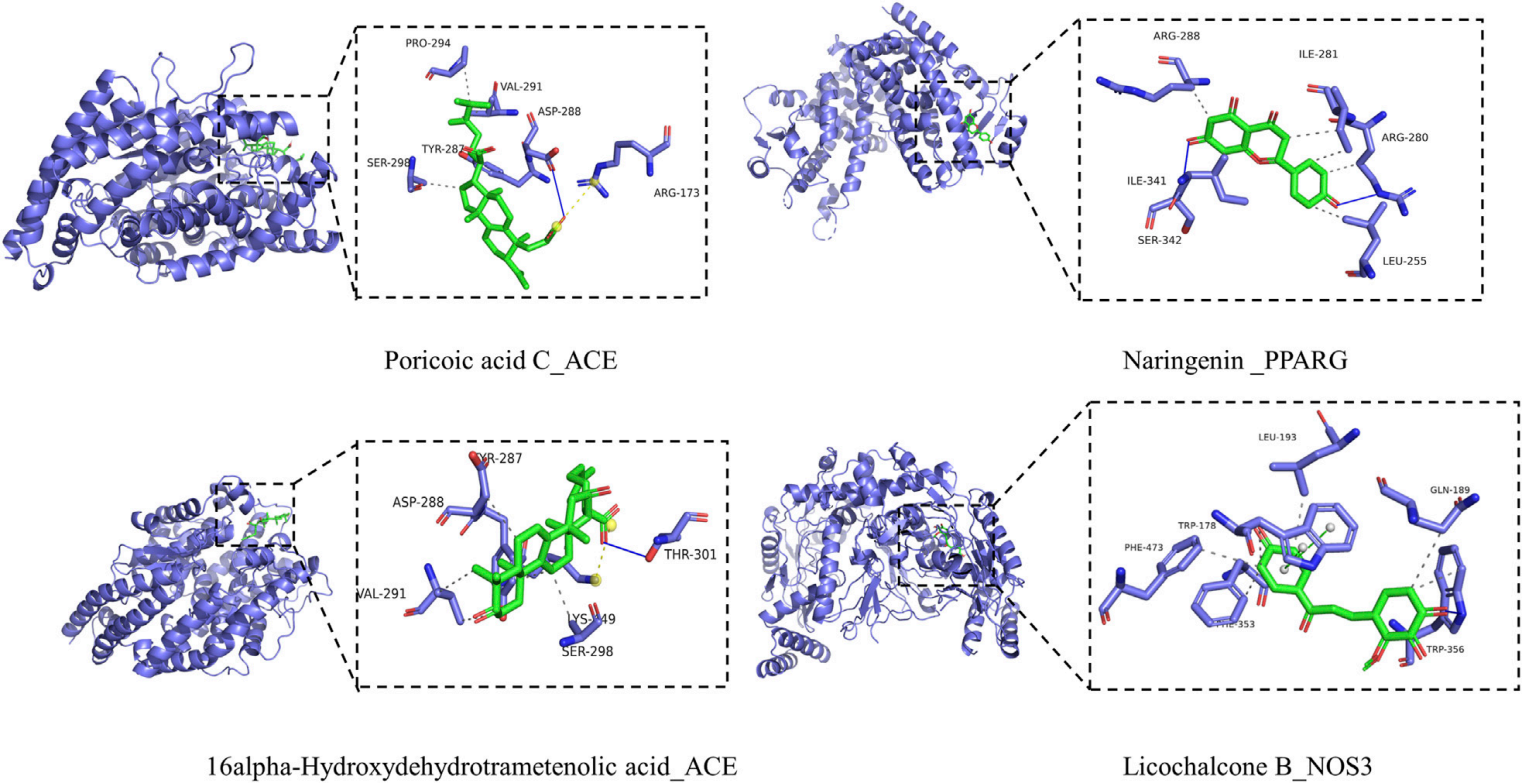
Molecular docking of key targets and key ingredients.

Through integrating data of Sections 3.5, 3.6, selected AKT1 (PDBID:1unp), NOS3 (PDBID:1m9j), ACE (PDBID: 1o86), and PPARG (PDBID:1i7i) as molecular docking targets, selected naringenin (CID: 932), 16alpha-Hydroxydehydrotrametenolic acid (CID: 10743008), poricoic acid C (CID:56668247), eburicoic acid (CID: 73402), licochalcone B (CID: 5318999) as binding ligands. The results showed that the docking energy was ≤−6 kcal·mol^−1^ (Table 5) and the receptor and ligand bind stably. Pymol and PLP were used to draw the result of molecular docking (Figure 13).

**TABLE 5:** Docking energy of the active ingredients and targets (kcal·mol^−1^).

Compounds	AKT1	NOS3	ACE	PPARG
Naringenin	−6.8	−9.4	−8.1	−7.6
16alpha-Hydroxydehydrotrametenolic acid	−7.3	−9.2	−8.6	−8.2
Poricoic acid C	−6.8	−9.3	−7.7	−7.6
Eburicoic acid	−6.8	−9.2	−7.8	−8.3
Licochalcone B	−6.4	−8.8	−7.7	−7.4

## 4 Discussion

To the best of our knowledge, this is the first article that integrates meta-analysis and network pharmacology to evaluate the efficacy and potential pharmacological mechanisms of BXD in the treatment of hypertension. This article reviews 23 intervention studies of BXD combined with conventional treatment in patients with hypertension, aiming to reveal the clinical effects of BXD. The meta-analysis found that compared with conventional treatment, combined BXD treatment was beneficial to improve clinical efficacy rate, blood pressure, blood lipids, Hcy, endothelial function, and inflammation. Notably, the combined treatment with BXD was effective in improving TCM symptoms which were important for improving the quality of life of patients. The treatment of hypertension should not only lower blood pressure but also improve the patient’s symptoms. According to TCM theory, the appearance of symptoms is a response to the imbalance of the internal environment of the body. Through the treatment of BXD, the internal environment of the body can be changed to improve the disease. In addition, BXD is safe and has no obvious adverse reactions. In general, BXD can be used as a complementary and alternative therapy for patients with hypertension on the premise of TCM syndrome differentiation.

Unfortunately, the overall quality of this study was not high. The methodological quality of RCTs was low, and the blinding, selective reporting of results and other biases were not described. In addition, the sample size of these RCTs was small, and they were all Chinese studies, and they were positive publications, which suggested a significant publication bias.

The pathological mechanism of hypertension is associated with endothelial dysfunction, increased vasoconstriction, and vascular changes characterized by arterial remodeling. The sympathetic nervous system, the renin-angiotensin-aldosterone system, and the immune system are all involved in the pathogenesis of hypertension (Coffman, 2011; Touyz et al., 2018). Using network pharmacology, we predicted the molecular mechanism of BXD against hypertension. The results showed that the key of BXD active ingredients including naringenin, 16 alpha-Hydroxydehydrotrametenolic acid, poricoic acid C, eburicoic acid, and licochalcone B. Naringenin is a flavanone, aglycone of naringin, exhibits a plethora of pharmacological properties. Studies have shown that naringenin exerts antihypertensive effects by attenuating the MCR/ACE/KIM-1 pathway (Oyagbemi et al., 2020). Recently, Liu et al. found that naringenin can reduce weight, fat, and blood pressure in obesity-associated hypertension rats, and the mechanism is related to the regulation of lipid disorders and oxidative stress (Liu et al., 2022). Eburicoic acid is present in the polyporus. Eburicoic acid has therapeutic potential for hyperlipidemia because it reduces adipose expression levels of lipogenic FAS and PPARγ, resulting in reduced lipolipid accumulation (Lin et al., 2017). Licochalcone B is a flavonoid active ingredient found in *Glycyrrhiza uralensis* Fisch. ex DC. [Fabaceae], which has a strong anti-inflammatory, antioxidant capacity, and can inhibit the production of NO, IL-6, and PGE2 in LPS-induced macrophage cells (Fu et al., 2013). Recent studies have shown that licochalcone B is also a specific NLRP3 (Li X et al., 2022). In conclusion, the active ingredients of BXD have potential pharmacological effects, and the beneficial effects of the active ingredients of BXD will be gradually explored with the development of technology.

In addition, we also found that the key antihypertensive targets of BXD were AKT1, NOS3, ACE, PPARG, TNF, and PTGS2. AKT regulates cell proliferation and growth and is involved in cellular processes including apoptosis and glucose metabolism. A study showed that AKT regulated endothelial function in SHR rats (Iaccarino et al., 2004). Cid-Soto et al. investigated the association of the eighth single nucleotide polymorphism in the AKT1 gene with different metabolic traits and found that AKT1 was associated with hypertension in Mestizos (Cid-Soto et al., 2018). NOS3 is important for vasodilation and heart rate (eNOS encoded by the NOS3), and eNOS knockout causes an increase in blood pressure (Shesely et al., 1996). Targeted disruption of the NOS gene in mice has become a useful tool to study cardiovascular endothelial dysfunction, response to vascular injury, and ischemia-reperfusion or atherosclerosis (Rochette et al., 2013). PPARG is a transcription factor that plays an important role in adipocyte differentiation, which is closely related to cardiometabolic diseases. A meta-analysis suggested that PPARG gene polymorphisms may be associated with the risk of hypertension (Cai et al., 2017). Similarly, Li et al. found that PPARG may also be involved in folic acid treatment of H-type hypertension (Liang et al., 2022). Subsequently, we performed validation by molecular docking and the results showed good affinity of the ligand and receptor. In addition, the possibility of combining targets and ingredients was further demonstrated by the literature review. For example, Liao et al. (2014) found demonstrated that naringenin could act by down-regulating AKT, and similarly, Zhang et al. (2015) found that naringenin inhibited the PI3K/AKT pathway, which in turn improved left ventricular function in pressure overload mice. Furthermore, it has been shown that naringenin treatment restored the impaired endothelium-dependent vasodilation by significantly increasing eNOS activity and NO levels. It is undeniable that the results of molecular docking still need to be verified by more experiments (Qin et al., 2016).

Finally, we also performed gene enrichment analysis for these targets. We found that these genes were mainly enriched in HIF-1 signaling pathway, fluid shear stress and atherosclerosis, calcium signaling pathway, cGMP-PKG signaling pathway. HIF-1 signaling pathway regulates oxygen homeostasis and plays an important role in the circulatory system. Evidence suggested that transcriptional changes in HIFs are an important molecular mechanism of hypertension (Semenza, 2014). Studies from Cowburn et al. found that the balance between HIF-1α and HIF-2α expression is a potential mechanism for the body to control blood pressure. They found that HIFs modulate macrophage production of NO *via* iNOS/NOS2 and arginase 1 (Takeda et al., 2010; Cowburn et al., 2013). Intracellular calcium signaling plays a crucial role in cardiovascular activity. The production of endothelium-derived vasoactive factors and the activation of endothelial potassium channels require elevated intracellular Ca^2+^levels. Disruption of Ca^2+^signaling circuits may contribute to endothelial dysfunction in hypertension (Sonkusare et al., 2014; Wilson et al., 2019). In summary, the above evidence suggested that BXD has the characteristics of regulating multiple pathways and multiple targets.

## 5 Conclusion

In conclusion, meta-analysis indicated that BXD combined with conventional treatment for hypertension is effective and safe. BXD has the characteristics of multi-pathway, multi-component, and multi-target in the treatment of hypertension. The antihypertensive targets of BXD may be AKT1, NOS3, ACE, and PPARG. The antihypertensive active ingredients of BXD may be naringenin, poricoic acid C, eburicoic acid, and licochalcone B. However, the evidence of BXD for hypertension should be carefully interpreted due to the low methodological quality, small sample size, limited number of trials, and other unidentified risks of bias. The efficacy and safety of BXD for hypertension still need to be further proved by high-quality clinical and basic studies.

## Data Availability

The original contributions presented in the study are included in the article/Supplementary Material, further inquiries can be directed to the corresponding author.
