# Supplementary material for: Banxia baizhu tianma decoction, a Chinese herbal formula, for hypertension: Integrating meta-analysis and network pharmacology
Source: Front Pharmacol. 2022 Dec 2;13:1025104. doi: 10.3389/fphar.2022.1025104 (PMC9755740; doi:10.3389/fphar.2022.1025104)
Supplement: Supplementary file 1 [file DataSheet1.docx]

Supplementary Material

**Supplementary Table 1**. The search strategy

| **Database** | **PubMed/Medline** | **Cochrane** | **Embase** |
| --- | --- | --- | --- |
| **Search term** | (((Hypertension [MeSH Terms]) OR (Essential Hypertension [Title/Abstract])) OR (high blood pressure [Title/Abstract])) AND ((((banxia baizhu tianma decoction [Title/Abstract]) OR (banxia baizhu Tiana[Title/Abstract])) OR (BXBZTM[Title/Abstract])) OR (BXBZ[Title/Abstract])) | "#1 - (hypertension): ti,ab,kw OR (high blood pressure):ti,ab,kw OR (Essential Hypertension):ti,ab,kw"  "#2- (banxia baizhu tianma decoction):ti,ab,kw OR (banxia baizhu tianma):ti,ab,kw OR (BXBZTM):ti,ab,kw OR (BXBZ):ti,ab,kw"  "#3 - #1 AND #2" | #1  hypertension:ti,ab,kw OR 'essential hypertension': ti,ab,kw OR 'high blood pressure': ti,ab,kw  #2  'banxia baizhu tianma’: ti,ab,kw OR bxbztm:ti,ab,kw OR bxbz:ti,ab,kw OR 'banxia baizhu tianma decoction':ti,ab,kw  #1 AND #2 |
| **Search record** | 7 | 15 | 9 |

**Supplementary Table 2**. The composition of the prescriptions

| **Study** | **Species, concentration** | **Quality control reported? (Y/N)** | **Chemical analysis reported? (Y/N)** |
| --- | --- | --- | --- |
| Dai HX 2022(Dai et al., 2022) | Tuber of *Pinellia ternata* (Thunb.) Makino, 20g Rhizome of *Atractylodes macrocephala* Koidz.,15g Pericarp of *Citrus* × *aurantium* L., 10g Root of *Glycyrrhiza uralensis* Fisch. ex DC., 10g Tuber of *Gastrodia elata* Blume, 20g, *Wolfiporia cocos* (F.A. Wolf) Ryvarden & Gilb.1984, 15g | N | N |
| Zhang W 2022(Zhang et al., 2022) | Tuber of *Pinellia ternata* (Thunb.) Makino, 10g Rhizome of *Atractylodes macrocephala* Koidz., 15g Pericarp of *Citrus* × *aurantium* L., 9g Root of *Glycyrrhiza uralensis* Fisch. ex DC., 6g Tuber of *Gastrodia elata* Blume, 10g, *Wolfiporia cocos* (F.A. Wolf) Ryvarden & Gilb.1984, 12g Fruit of *Ziziphus jujuba* Mill., 6g Rhizome of *Zingiber officinale* Roscoe, 5g | N | N |
| Zhao XD 2022(Zhao, 2022) | Tuber of *Pinellia ternata* (Thunb.) Makino, 10g Rhizome of *Atractylodes macrocephala* Koidz., 15g Pericarp of *Citrus* × *aurantium* L., 6g Root of *Glycyrrhiza uralensis* Fisch. ex DC., 6g Tuber of *Gastrodia elata* Blume, 10g, *Wolfiporia cocos* (F.A. Wolf) Ryvarden & Gilb.1984, 15g Fruit of *Ziziphus jujuba* Mill., 6g Rhizome of *Zingiber officinale* Roscoe, 5g Rhizome of *Alisma plantago-aquatica* L., 20g Rhizome of *Conioselinum anthriscoides 'Chuanxiong'*, 6g Fruit of *Crataegus pinnatifida* Bunge, 12g | N | N |
| Zhang Y 2021(Zhang, 2021) | Tuber of *Pinellia ternata* (Thunb.) Makino, 9g Rhizome of *Atractylodes macrocephala* Koidz., 15g Pericarp of *Citrus* × *aurantium* L., 10g Root of *Glycyrrhiza uralensis* Fisch. ex DC., 4g Tuber of *Gastrodia elata* Blume, 10g, *Wolfiporia cocos* (F.A. Wolf) Ryvarden & Gilb.1984, 10g Rhizome of *Conioselinum anthriscoides 'Chuanxiong'*, 9g Rhizome of *Alisma plantago-aquatica* L., 15g Rhizome of *Acorus gramineus* Aiton, 10g Fruit of *Ziziphus jujuba* Mill., 6g Rhizome of *Zingiber officinale* Roscoe, 5g Polyporus umbellatus (Pers.) Fr., 10g | N | N |
| Zheng XL 2021(Zheng, 2021) | Tuber of *Pinellia ternata* (Thunb.) Makino, 10g Rhizome of *Atractylodes macrocephala* Koidz., 10g Pericarp of *Citrus* × *aurantium* L., 10g Root of *Glycyrrhiza uralensis* Fisch. ex DC., 5g Tuber of *Gastrodia elata* Blume, 10g, *Wolfiporia cocos* (F.A. Wolf) Ryvarden & Gilb.1984, 10g Fruit of *Ziziphus jujuba* Mill., 6g Rhizome of *Zingiber officinale* Roscoe, 5g | N | N |
| Zhang QQ 2021(Zhang et al., 2021) | Tuber of *Pinellia ternata* (Thunb.) Makino, 15g Rhizome of *Atractylodes macrocephala* Koidz., 12g Pericarp of *Citrus* × *aurantium* L., 10g Root of *Glycyrrhiza uralensis* Fisch. ex DC., 9g Tuber of *Gastrodia elata* Blume, 10g, *Wolfiporia cocos* (F.A. Wolf) Ryvarden & Gilb.1984, 12g Fruit of *Ziziphus jujuba* Mill., 9g Rhizome of *Zingiber officinale* Roscoe, 9g Root of *Salvia miltiorrhiza* Bunge, 9g Tuber of *Corydalis yanhusuo* (Y.H.Chou & Chun C.Hsu) W.T.Wang ex Z.Y.Su & C.Y.Wu, 9g | N | N |
| Liu RX 2020(Liu et al., 2020) | Tuber of *Pinellia ternata* (Thunb.) Makino Rhizome of *Atractylodes macrocephala* Koidz. Pericarp of *Citrus* × *aurantium* L.  Root of *Glycyrrhiza uralensis* Fisch. ex DC. Tuber of *Gastrodia elata* Blume  *Wolfiporia cocos* (F.A. Wolf) Ryvarden & Gilb.1984 | N | N |
| Tang L 2020(Tang et al., 2020) | Tuber of *Pinellia ternata* (Thunb.) Makino, 9g Rhizome of *Atractylodes macrocephala* Koidz., 15g Pericarp of *Citrus* × *aurantium* L., 10g Root of *Glycyrrhiza uralensis* Fisch. ex DC., 6g Tuber of *Gastrodia elata* Blume, 10g, *Wolfiporia cocos* (F.A. Wolf) Ryvarden & Gilb.1984, 15g Rhizome of *Acorus gramineus* Aiton,15g Shavings of *Bambusa tuldoides* Munro, 10g Seed of *Ziziphus jujuba* Mill., 15g Fruit of *Citrus* × *aurantium* L., 10g Root of *Polygala tenuifolia* Willd., 10g | N | N |
| Mu SS 2020(Mu, 2020) | Tuber of *Pinellia ternata* (Thunb.) Makino, 10g Rhizome of *Atractylodes macrocephala* Koidz., 20g Pericarp of *Citrus* × *aurantium* L., 20g Root of *Glycyrrhiza uralensis* Fisch. ex DC., 6g Tuber of *Gastrodia elata* Blume, 30g, *Wolfiporia cocos* (F.A. Wolf) Ryvarden & Gilb.1984, 20g Rhizome of *Conioselinum anthriscoides 'Chuanxiong’,* 10g Fruit of *Vitex trifolia* L., 20g Root of *Pueraria montana var. lobata* (Willd.) Maesen & S.M.Almeida ex Sanjappa & Predeep, 30g Stem of *Uncaria rhynchophylla* (Miq.) Miq., 30g | N | N |
| Ma HN 2019(Ma et al., 2019) | Tuber of *Pinellia ternata* (Thunb.) Makino, 10g Rhizome of *Atractylodes macrocephala* Koidz., 15g Pericarp of *Citrus* × *aurantium* L., 6g Root of *Glycyrrhiza uralensis* Fisch. ex DC., 6g Tuber of *Gastrodia elata* Blume, 10g, *Wolfiporia cocos* (F.A. Wolf) Ryvarden & Gilb.1984, 10g Fruit of *Ziziphus jujuba* Mill., 10g Rhizome of *Zingiber officinale* Roscoe, 10g | N | N |
| Wu ZJ 2019(Wu, 2019) | Tuber of *Pinellia ternata* (Thunb.) Makino, 9g Rhizome of *Atractylodes macrocephala* Koidz., 9g Pericarp of *Citrus* × *aurantium* L., 6g Root of *Glycyrrhiza uralensis* Fisch. ex DC., 6g Tuber of *Gastrodia elata* Blume, 9g, *Wolfiporia cocos* (F.A. Wolf) Ryvarden & Gilb.1984, 9g Fruit of Ziziphus jujuba Mill., 6g Rhizome of *Zingiber officinale* Roscoe, 9g | N | N |
| Shi CZ 2019(Shi, 2019) | Tuber of *Pinellia ternata* (Thunb.) Makino, 15g Rhizome of *Atractylodes macrocephala* Koidz., 20g Pericarp of *Citrus* × *aurantium* L., 20g Root of *Glycyrrhiza uralensis* Fisch. ex DC., 10g Tuber of *Gastrodia elata* Blume, 20g, *Wolfiporia cocos* (F.A. Wolf) Ryvarden & Gilb.1984, 15g | N | N |
| Song GP 2018(Song, 2018) | Tuber of *Pinellia ternata* (Thunb.) Makino, 10g Rhizome of *Atractylodes macrocephala* Koidz., 20g Pericarp of *Citrus* × *aurantium* L., 10g Root of *Glycyrrhiza uralensis* Fisch. ex DC., 6g Tuber of *Gastrodia elata* Blume, 10g, *Wolfiporia cocos* (F.A. Wolf) Ryvarden & Gilb.1984, 10g Stem of *Reynoutria multiflora* (Thunb.) Moldenke, 10g | N | N |
| Miao LJ 2017(Miao et al., 2017) | Tuber of *Pinellia ternata* (Thunb.) Makino, 9g Rhizome of *Atractylodes macrocephala* Koidz., 15g Pericarp of *Citrus* × *aurantium* L., 10g Root of *Glycyrrhiza uralensis* Fisch. ex DC., 5g Tuber of *Gastrodia elata* Blume, 6g, *Wolfiporia cocos* (F.A. Wolf) Ryvarden & Gilb.1984, 6g Tuber of *Alisma plantago-aquatica* L*.*, 6g Root of *Paeonia lactiflora* Pall., 10g Leaf of *Nelumbo nucifera* Gaertn., 15g | N | N |
| Zhao HY 2016(Zhao et al., 2016) | Tuber of *Pinellia ternata* (Thunb.) Makino, 4.5g Rhizome of *Atractylodes macrocephala* Koidz., 9g Pericarp of *Citrus* × *aurantium* L., 15g Root of *Glycyrrhiza uralensis* Fisch. ex DC., 4.5g Tuber of *Gastrodia elata* Blume, 15g, *Wolfiporia cocos* (F.A. Wolf) Ryvarden & Gilb.1984, 15g Fruit of Ziziphus jujuba Mill., 10g Rhizome of *Zingiber officinale* Roscoe, 3g | N | N |
| Guan JL 2016(Guan and Chen, 2016) | Tuber of *Pinellia ternata* (Thunb.) Makino, 10g Rhizome of *Atractylodes macrocephala* Koidz., 15g Pericarp of *Citrus* × *aurantium* L., 6g Root of *Glycyrrhiza uralensis* Fisch. ex DC., 6g Tuber of *Gastrodia elata* Blume, 10g, *Wolfiporia cocos* (F.A. Wolf) Ryvarden & Gilb.1984 , 15g Fruit of Ziziphus jujuba Mill., 6g Rhizome of *Zingiber officinale* Roscoe, 4g Bark of *Magnolia officinalis* Rehder & E.H.Wilson, 15g Fruit of *Crataegus pinnatifida* Bunge, 12g Root of  *Pueraria montana var. lobata* (Willd.) Maesen & S.M.Almeida ex Sanjappa & Predeep, 10g | N | N |
| Liu XL 2016(Liu, 2016) | Tuber of *Pinellia ternata* (Thunb.) Makino, 9g Rhizome of *Atractylodes macrocephala* Koidz., 9g Pericarp of *Citrus* × *aurantium* L, 6g Root of *Glycyrrhiza uralensis* Fisch. ex DC., 6g Tuber of *Gastrodia elata* Blume, 9g, *Wolfiporia cocos* (F.A. Wolf) Ryvarden & Gilb.1984, 9g Fruit of Ziziphus jujuba Mill., 9g Rhizome of *Zingiber officinale* Roscoe, 6g | N | N |
| Wu HH 2016(Wu and Zhou, 2016) | Tuber of *Pinellia ternata* (Thunb.) Makino, 10g Rhizome of *Atractylodes macrocephala* Koidz., 15g Pericarp of *Citrus* × *aurantium* L., 6g Root of *Glycyrrhiza uralensis* Fisch. ex DC., 6g Tuber of *Gastrodia elata* Blume, 10g, *Wolfiporia cocos* (F.A. Wolf) Ryvarden & Gilb.1984, 15g Rhizome of *Conioselinum anthriscoides 'Chuanxiong’,* 6g Root of Pueraria montana var. lobata (Willd.) Maesen & S.M.Almeida ex Sanjappa & Predeep, 10g Fruit of Crataegus pinnatifida Bunge, 12g | N | N |
| Shen QS 2015(Shen and Jin, 2015) | Tuber of *Pinellia ternata* (Thunb.) Makino, 10g Rhizome of *Atractylodes macrocephala* Koidz., 20g Pericarp of *Citrus* × *aurantium* L., 10g Root of *Glycyrrhiza uralensis* Fisch. ex DC., 6g Tuber of *Gastrodia elata* Blume, 10g, *Wolfiporia cocos* (F.A. Wolf) Ryvarden & Gilb.1984, 10g Stem of *Reynoutria multiflora* (Thunb.) Moldenke, 10g | N | N |
| Huang ZS 2014(Huang and Li, 2014) | Tuber of *Pinellia ternata* (Thunb.) Makino, 15g Rhizome of *Atractylodes macrocephala* Koidz., 15g Pericarp of *Citrus* × *aurantium* L., 10g Root of *Glycyrrhiza uralensis* Fisch. ex DC., 6g Tuber of *Gastrodia elata* Blume, 10g, *Wolfiporia cocos* (F.A. Wolf) Ryvarden & Gilb.1984, 10g | N | N |
| Pang YH 2013(Pang, 2013) | Tuber of *Pinellia ternata* (Thunb.) Makino, 10g Rhizome of *Atractylodes macrocephala* Koidz., 15g Pericarp of *Citrus* × *aurantium* L.,6g Root of *Glycyrrhiza uralensis* Fisch. ex DC.,6g Tuber of *Gastrodia elata* Blume, 10g, *Wolfiporia cocos* (F.A. Wolf) Ryvarden & Gilb.1984, 15g Rhizome of *Conioselinum anthriscoides 'Chuanxiong’,* 6g Root of Pueraria montana var. lobata (Willd.) Maesen & S.M.Almeida ex Sanjappa & Predeep, 10g Fruit of Crataegus pinnatifida Bunge, 12g Rhizome of *Alisma plantago-aquatica* L., 20g | N | N |
| Xiong YW 2010(Xiong, 2010) | Tuber of *Pinellia ternata* (Thunb.) Makino, 12g Rhizome of *Atractylodes macrocephala* Koidz., 15g Pericarp of *Citrus* × *aurantium* L., 12g Root of *Glycyrrhiza uralensis* Fisch. ex DC., 6g Tuber of *Gastrodia elata* Blume, 15g, *Wolfiporia cocos* (F.A. Wolf) Ryvarden & Gilb.1984, 12g Fruit of Ziziphus jujuba Mill., 10g Rhizome of *Zingiber officinale* Roscoe, 9g Rhizome of *Alisma plantago-aquatica* L., 15g Tuber of *Arisaema heterophyllum* Blume, 12g Seed of *Plantago asiatica* L., 15g Rhizome of Acorus gramineus Aiton, 15g Shavings of Bambusa tuldoides Munro, 10g Fruit of *Wurfbainia villosa* (Lour.) Skornick. & A.D.Poulsen , 3g | N | N |
| Wu QF 2007(Wu et al., 2007) | Tuber of *Pinellia ternata* (Thunb.) Makino, 10g Rhizome of *Atractylodes macrocephala* Koidz., 10g Pericarp of *Citrus* × *aurantium* L., 10g Root of *Glycyrrhiza uralensis* Fisch. ex DC., 3g Tuber of *Gastrodia elata* Blume, 10g, *Wolfiporia cocos* (F.A. Wolf) Ryvarden & Gilb.1984, 15g Rhizome of *Zingiber officinale* Roscoe, 6g Shavings of Bambusa tuldoides Munro, 10g Seed of *Coix lacryma-jobi* L., 20g | N | N |


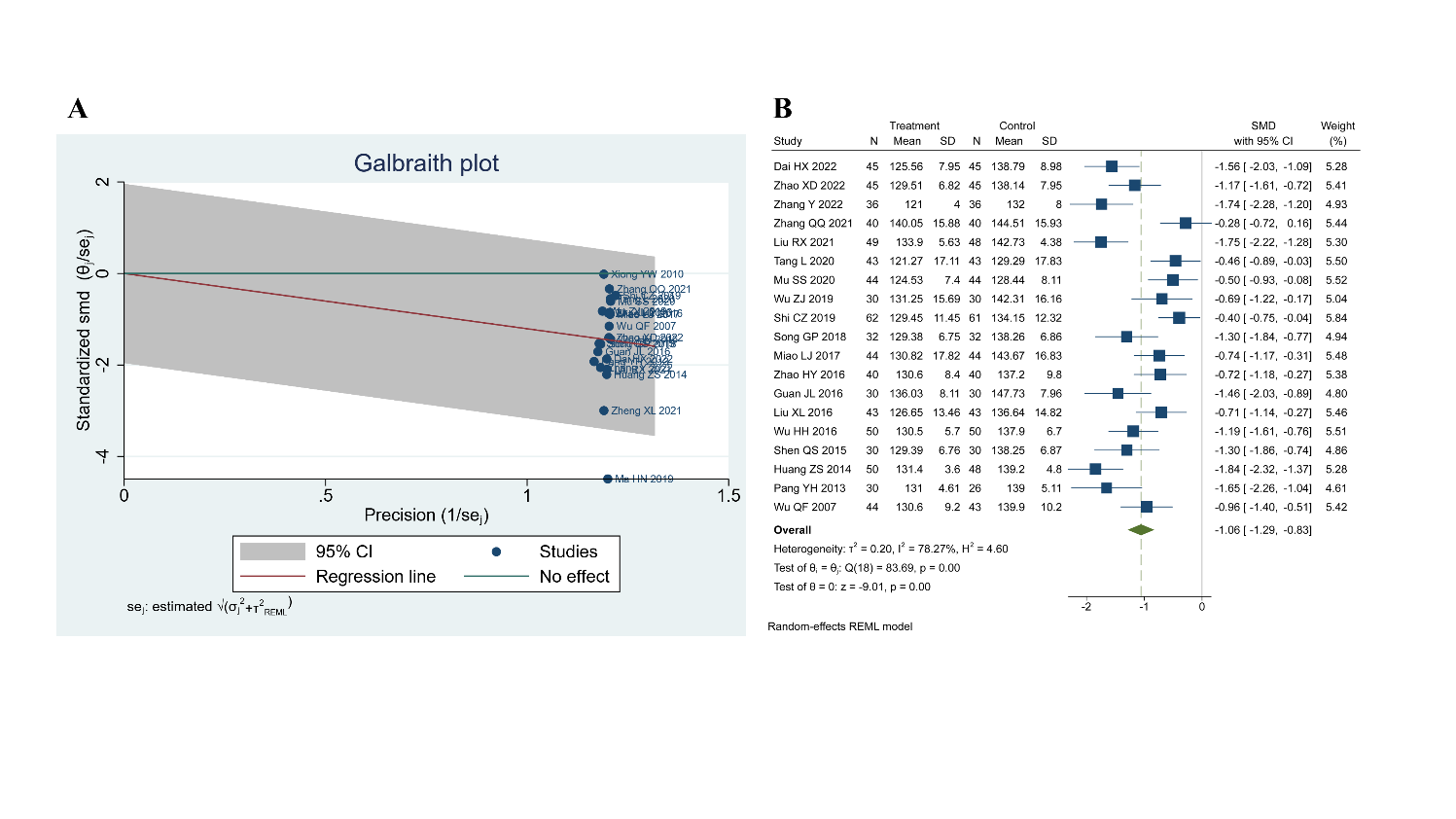


**Figure S1.** Sensitivity analysis of SBP (A) Galbraith plot of SBP; (B) Forest plots after exclusion of heterogeneity studies.


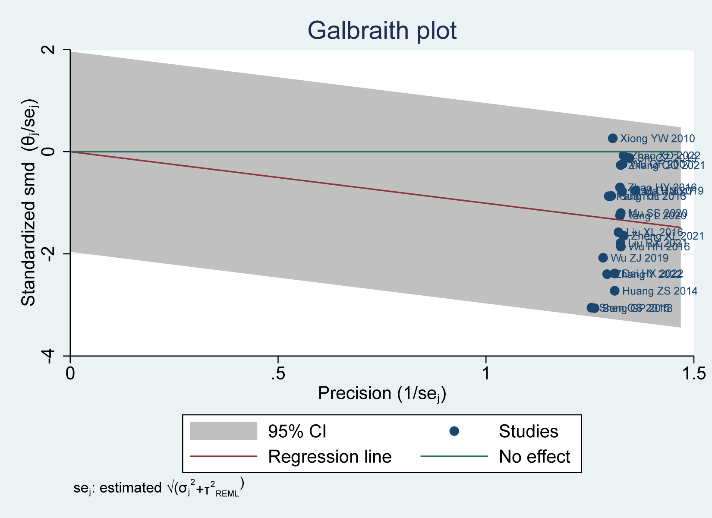


**Figure S2.** Galbraith plot of DBP.


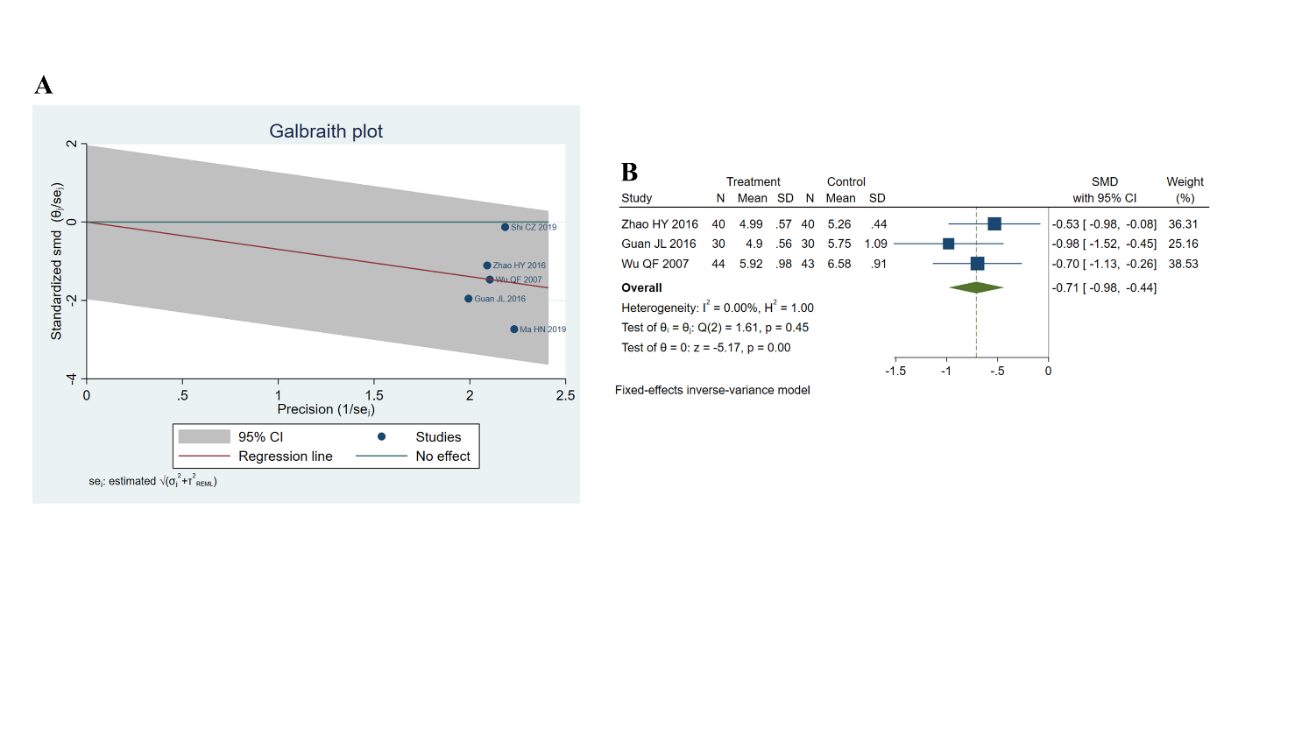


**Figure S3.** Sensitivity analysis of TC (A) Galbraith plot of TC; (B) Forest plots after exclusion of heterogeneity studies.


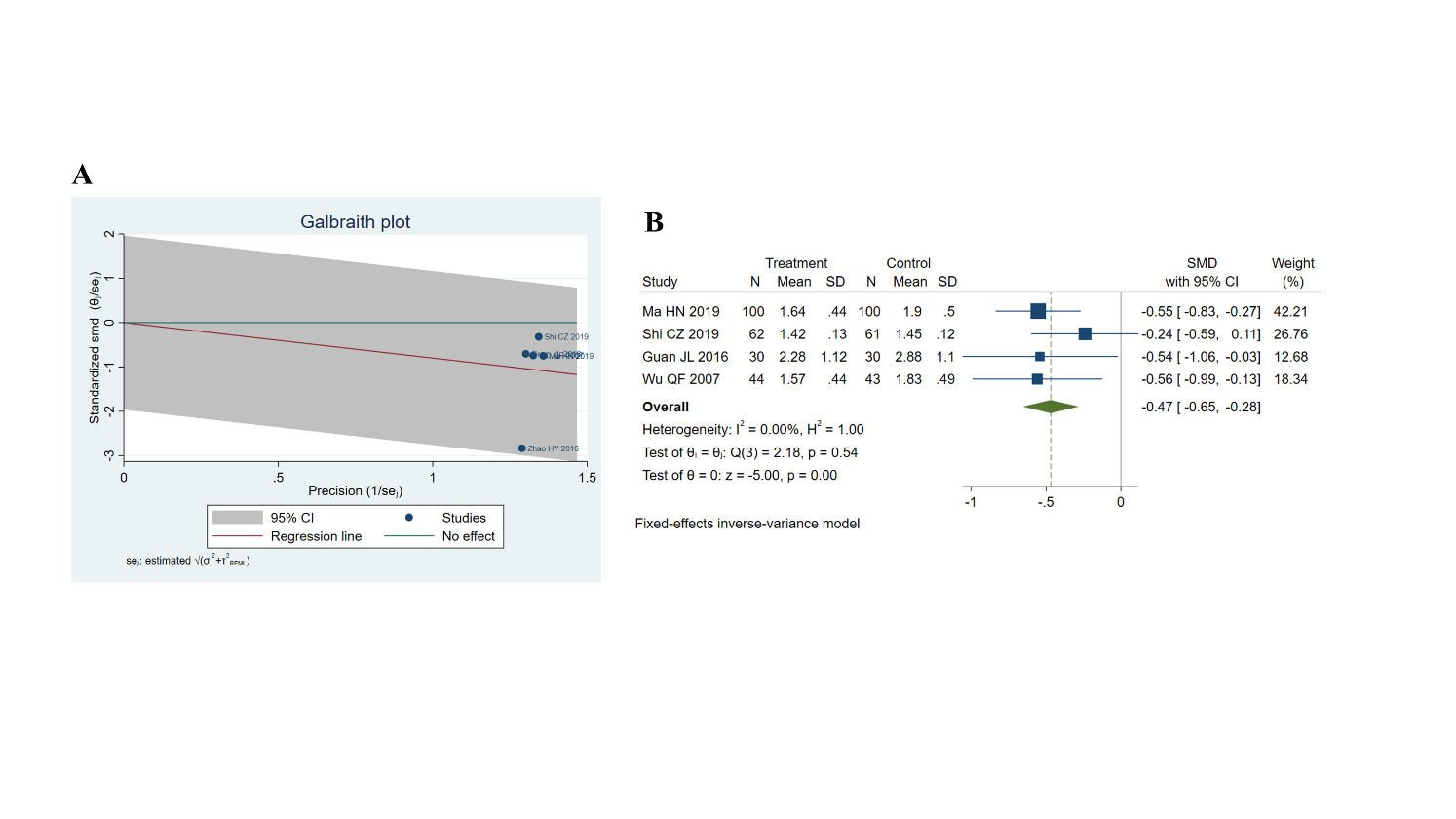


**Figure S4.** Sensitivity analysis of TG (A) Galbraith plot of TG; (B) Forest plots after exclusion of heterogeneity studies.


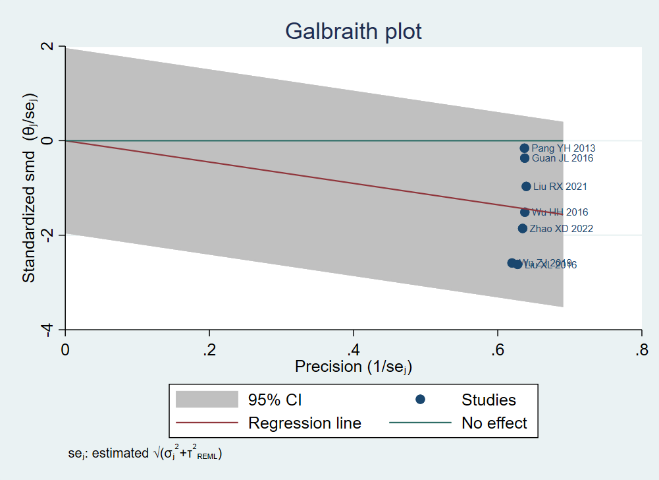


**Figure S5.** Galbraith plot of Hcy.


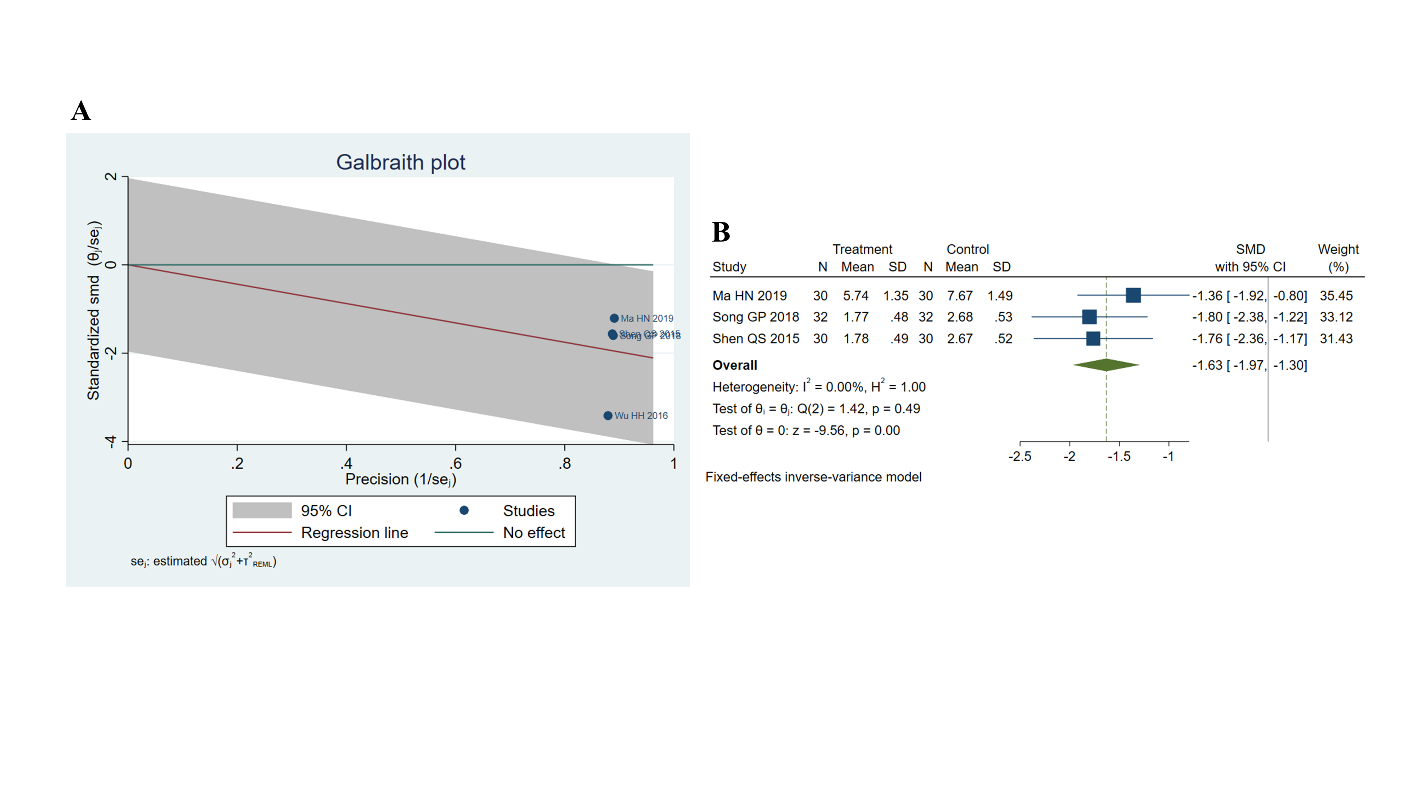


**Figure S6.** Sensitivity analysis of CRP (A) Galbraith plot of CRP; (B) Forest plots after exclusion of heterogeneity studies.


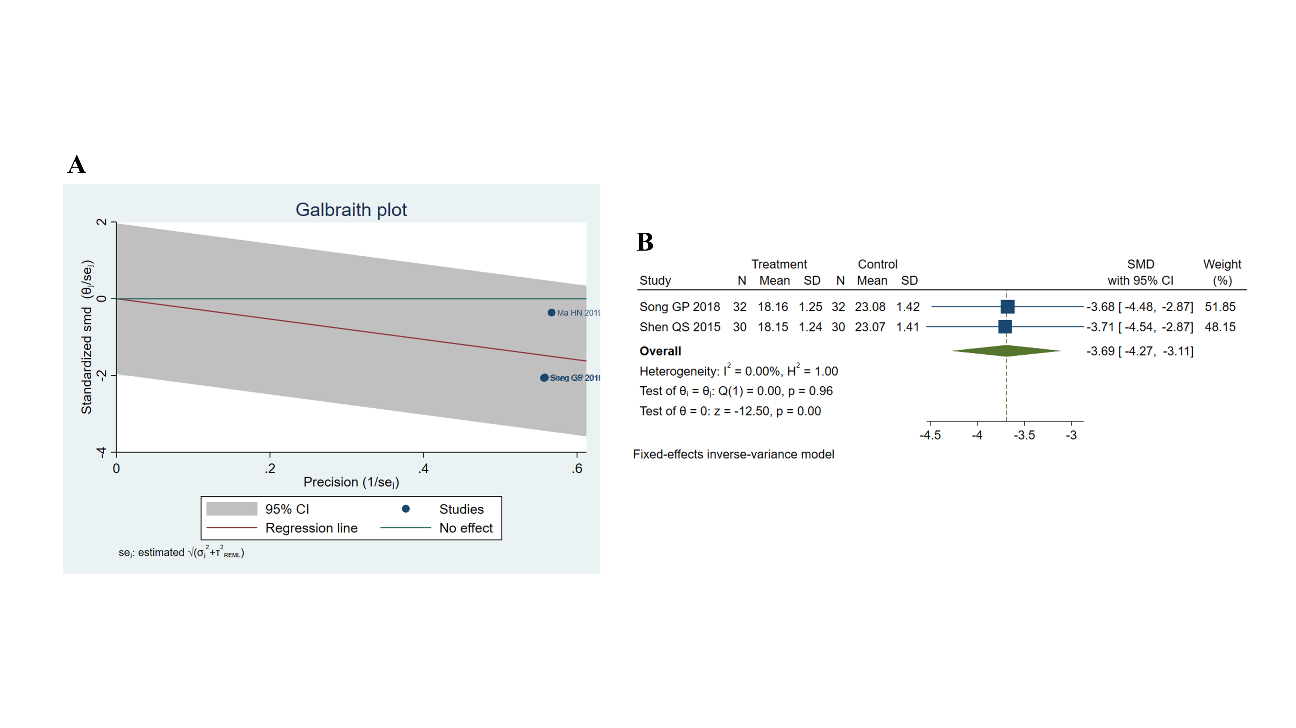


**Figure S7.** Sensitivity analysis of IL-6 (A) Galbraith plot of IL-6; (B) Forest plots after exclusion of heterogeneity studies.


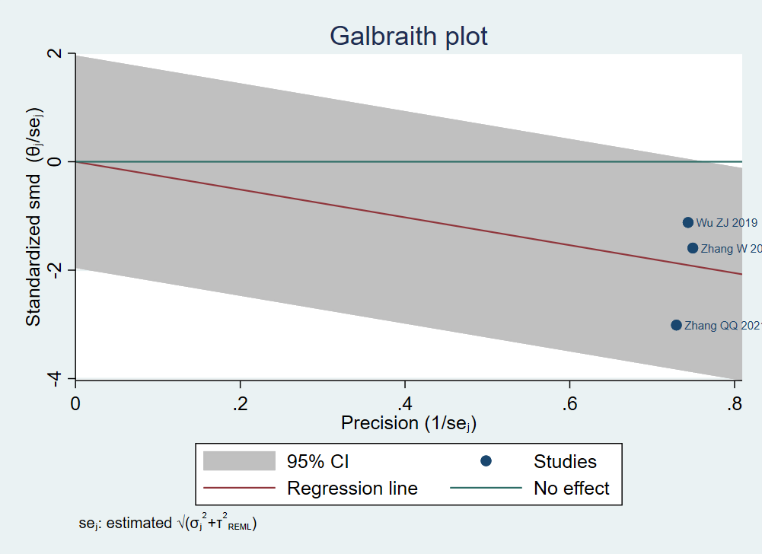


**Figure S8.** Galbraith plot of vertigo.


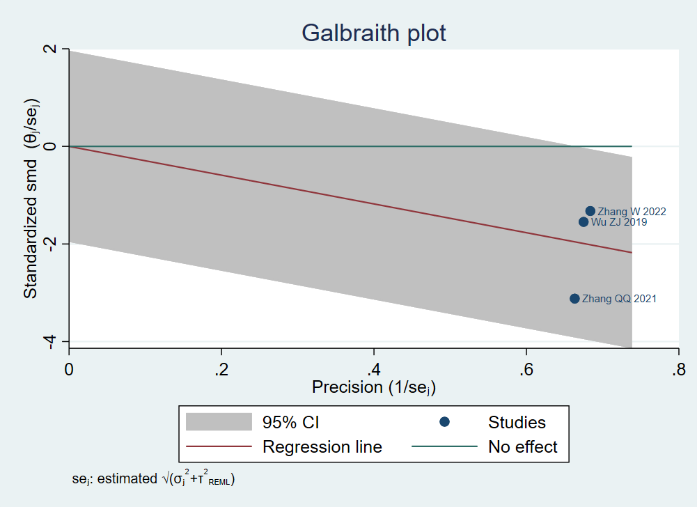


**Figure S9.** Galbraith plot of anorexia.


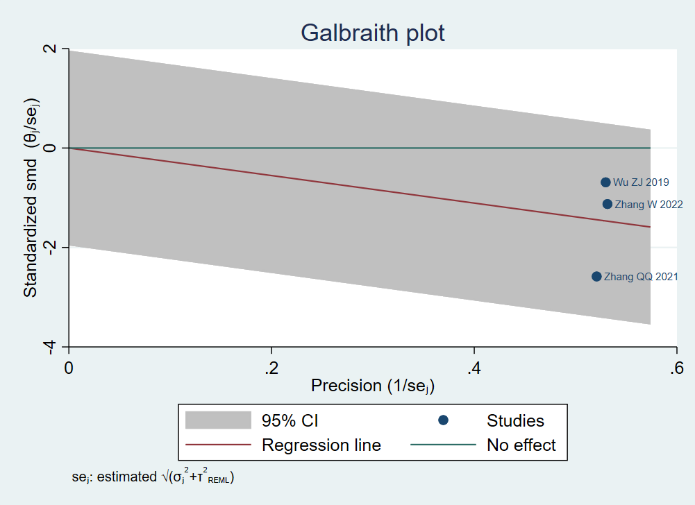


**Figure S10.** Galbraith plot of chest tightness and fatigue.
